# Current and future burden trends of lip and oral cavity cancer based on estimated annual percentage change and the Bayesian age-period-cohort model: a study based on the global burden of disease 2021

**DOI:** 10.3389/fonc.2025.1674054

**Published:** 2025-10-22

**Authors:** Qiong-Ying Mu, Min-Li, Si-Wen Chen, Sheng-Rong Tang

**Affiliations:** Stomatology Department, The First People's Hospital of Yulin (The Sixth Affiliated Hospital of Guangxi Medical University), Yulin, China

**Keywords:** lip and oral cavity cancer, global burden of disease, estimated annual percentage change, bayesian age-period-cohort model, prevention

## Abstract

**Introduction:**

Lip and Oral Cavity Cancer (LOCC), as a common type of malignant cancer, has become an important public health issue. This study aims to analyze and predict the trend of the LOCC burden, providing guidance for reducing the burden of LOCC.

**Methods:**

This study was based on the Global Burden of Disease (GBD) database 2021. We assessed the differences in LOCC among different regions and sexes from 1990 to 2021 using Age-Standardized Rates (ASR) and Estimated Annual Percentage Change (EAPC). Additionally, Bayesian Age-Period-Cohort (BAPC) model was employed to predict the Incidence, Prevalence, Death, Disability-Adjusted Life Years (DALYs), Years of Life Lost due to disease (YLLs), and Years Lived with Disability (YLDs) of LOCC globally from 2022 to 2030.

**Results:**

From 1990 to 2021, the global ASR of Incidence (ASIR), ASR of Prevalence (ASPR), and ASR of YLDs for LOCC had significantly increased, while the ASR of Death (ASDR), DALYs, and YLLs had decreased. The BAPC model predicted that by 2030, the ASIR and ASPR for males would decrease, while for females, an upward trend would be observed. The middle-aged and elderly population (>50 years) would still be the main patient group (accounting for over 60%).

**Conclusion:**

The disease burden of LOCC remains severe at present, but it is expected to show a downward trend by 2030. Therefore, it is still necessary to intensify efforts in the prevention and control of LOCC to achieve the expectation. This study provides theoretical guidance for reducing the burden of LOCC.

## Introduction

Lip and oral cavity cancer (LOCC) is a malignant cancer involving the lips, tongue, gums, oral mucosa, and salivary glands, and is one of the common cancers worldwide (1). The severe impact of LOCC on patients’ speech, eating, and overall quality of life makes it an important public health issue (2). Studies have shown that smoking, alcohol consumption, chewtabaccio, betelnut, and long-term ultraviolet (UV) exposure are all significant risk factors for LOCC (3, 4). With the intensification of global population aging and the widespread presence of these risk factors, the situation of LOCC remains severe (5). Specifically, in some low- and middle-income countries, the diagnosis of LOCC is often delayed due to the lack of effective early screening and treatment methods, which leads to increased difficulty in treatment and poor prognosis (6).

Beyond its impact on the physical health of LOCC patients, it exerts a substantial economic burden on different regions. For instance, in Iran, the overall economic burden attributable to oral cancer reaches 64,245,173 USD, with the cost of late-stage treatment being five times higher than that of early-stage treatment (7). Similarly, in Australia, the average economic burden per patient with oral squamous cell carcinoma amounts to 61,128 USD (8). Therefore, understanding the current and future trends of LOCC disease burden is crucial for reducing, controlling, and preventing LOCC. It can serve as a guide for policymakers in planning and implementing effective prevention and control programs.

Based on this, our study aims to leverage the data from the Global Burden of Disease (GBD) 2021 to stratify the current and future trends in the global burden of LOCC, and to investigate the differences across genders and age groups. This endeavor is intended to provide a scientific basis for LOCC prevention and control strategies on a global level. Through the analysis of LOCC burden, we hope to offer valuable insights to public health policymakers, thereby promoting global efforts in the prevention, early diagnosis, and treatment of LOCC, and ultimately alleviating the burden of LOCC on human health.

## Methods

### Data source and selection

The data on LOCC in this study were sourced from the GBD 2021 database (https://vizhub.healthdata.org/gbd-results/). This database integrates data from multiple sources and provides detailed epidemiological data for 195 countries and regions worldwide, covering 359 diseases, injuries, and risk factors (9, 10). Moreover, the data utilized in this study were derived from publicly accessible datasets that have been endorsed by the University of Washington, thereby eliminating any ethical concerns (9, 10). Finally, this study focused on analyzing the current status and future projections of LOCC based on the current data, including Incidence, Prevalence, Death, Disability-Adjusted Life Years (DALYs), Years of Life Lost (YLLs) due to premature mortality, and Years Lived with Disability (YLDs).

### Data processing

To characterize the LOCC burden in 2021, this study utilized Age-Standardized Rate (ASR) to mitigate the impact of population age structure, thereby enhancing the comparability of LOCC burden across different regions (9). The precision of ASR estimates was further quantified using 95% Uncertainty Intervals (UI) (9). Additionally, to assess the trend in the burden of LOCC from 1990 to 2021, the Estimate Annual Percentage Change (EAPC) was employed. EAPC is a statistical measure that quantifies the change in disease incidence or mortality over time, calculated using the formula 100×(*exp(β)*−1), where *β* represents the coefficient of the time variable in a linear regression model. The EAPC results provide the average annual percentage change, with positive values indicating an increase over time and negative values indicating a decrease (11, 12). The 95% Confidence Interval (CI) reflects the uncertainty around the EAPC estimate typically (11, 12).

Subsequently, to explore the disease burden trends of LOCC in the next nine years, we employed the Bayesian age-period-cohort (BAPC) model to project the LOCC burden up to 2030. This model integrates three principal dimensions (age effects), period effects, and cohort effects. Utilizing a Bayesian statistical framework, the BAPC model allows researchers to incorporate prior knowledge and quantify uncertainty within the model. The Bayesian approach, by combining data information with prior distributions to generate posterior distributions, provides more precise parameter estimates. The BAPC model not only describes historical trends but also predicts future disease burden. Compared with traditional epidemiological models, the BAPC model offers a more comprehensive consideration of the various factors influencing disease trends, providing a more detailed and dynamic view of disease burden. Additionally, the Bayesian nature of the model enables the quantification of uncertainty, which enhances the interpretability and credibility of the results (13, 14).

## Results

### The burden of LOCC from 1990 to 2021

Globally, compared to 1990, the number of LOCC cases or years in terms of Incidence, Prevalence, Death, DALYs, YLLs, and YLDs had increased several-fold in 2021. However, the number only provides a rudimentary snapshot of the LOCC burden at different time points. Therefore, this study employed ASR to compare the current burden of LOCC across different regions. In 2021, the age-standardized prevalence rate (ASPR) and the age-standardized rate of YLDs were highest in Australasia, with High-income North America and South Asia following closely behind. However, the highest age-standardized incidence rate (ASIR), age-standardized death rate (ASDR), age-standardized rate of DALYs, and YLLs were all observed in South Asia. Focusing solely on 2021, LOCC patients in three regions, including Australasia, had longer survival durations and later time points of death. Notably, the ASDR in South Asia was more than four times that of Australasia. Thus, the burden of LOCC in South Asia remains the most severe (**Table 1**–**6**).

**Table 1:** Changes in lip and oral cavity cancer Incidence by regions and sexes.

Locations	1990 Number (95% UI, cases)	1990 Age-standardized rate (95% UI, per 100,000)	2021 Number (95% UI, cases)	2021 Age-standardized rate (95% UI, per 100,000)	Estimated annual percentage change (95% CI, 100%)
Both sexes	Incidence				
Global	174077 (167404-181621)	4.3 (4.1-4.5)	421577 (389878-449782)	4.9 (4.5-5.2)	0.3981 (0.3332-0.4631)
High-middle SDI	36685 (35237-38039)	3.6 (3.5-3.8)	75381 (68753.5-81578)	3.9 (3.5-4.2)	0.1374 (0.044-0.2308)
High SDI	58203 (56115-59655)	5.4 (5.3-5.6)	104871 (97792-109602)	5.4 (5.1-5.7)	0.0629 (0.0028-0.1231)
Low-middle SDI	35520 (31899-39325)	5.4 (4.9-6.0)	102039 (90115-113221)	6.7 (6.0-7.5)	0.6426 (0.559-0.7263)
Low SDI	9501 (8202-10847)	3.9 (3.4-4.4)	25089 (21421-28947)	4.6 (3.9-5.2)	0.3313 (0.2285-0.4342)
Middle SDI	33981 (31865-36099)	3.1 (2.9-3.3)	113857 (102889-125357)	4.2 (3.8-4.6)	0.8903 (0.7929-0.9877)
Andean Latin America	283 (247-322)	1.3 (1.2-1.5)	945 (752-1166)	1.6 (1.3-1.9)	0.6085 (0.4407-0.7766)
Australasia	1692 (1555-1832)	7.3 (6.7-7.9)	3469 (3088-3840)	7.0 (6.3-7.7)	-0.1487 (-0.3425-0.0453)
Caribbean	1069 (1002-1143)	4.1 (3.8-4.4)	2111 (1823-2406)	3.9 (3.4-4.5)	0.0733 (-0.0339-0.1806)
Central Asia	1264 (1176-1371)	2.6 (2.4-2.8)	2057 (1800-2354)	2.4 (2.1-2.7)	-0.1940 (-0.4477-0.0604)
Central Europe	6364 (6075-6683)	4.3 (4.1-4.5)	11404 (10501-12286)	5.6 (5.2-6.1)	0.8003 (0.6951-0.9056)
Central Latin America	1530 (1477-1576)	1.8 (1.8-1.9)	4442 (3946-4965)	1.8 (1.6-2.0)	-0.2814 (-0.3885--0.1743)
Central Sub-Saharan Africa	464 (357-601)	2.0 (1.6-2.5)	1307 (996-1674)	2.2 (1.7-2.8)	0.3127 (0.1506-0.475)
East Asia	16020 (13746-18301)	1.8 (1.5-2.0)	63514 (51900-77072)	2.9 (2.4-3.5)	1.8681 (1.7032-2.0333)
Eastern Europe	13201 (12692-13983)	4.7 (4.6-5.0)	21039 (19042-23091)	6.5 (5.9-7.1)	0.6676 (0.4681-0.8675)
Eastern Sub-Saharan Africa	2582 (2247-2946)	3.2 (2.8-3.7)	6336 (5060-7633)	3.4 (2.8-4.0)	0.0284 (-0.0526-0.1095)
High-income Asia Pacific	5917 (5642-6147)	2.9 (2.8-3.1)	18189 (15884-19823)	4.2 (3.8-4.5)	1.0902 (0.7726-1.4088)
High-income North America	24125 (23178-24789)	7.2 (6.9-7.4)	36565 (34331-38154)	5.9 (5.5-6.1)	-0.6355 (-0.7335--0.5374)
North Africa and Middle East	1836 (1615-2089)	1.1 (0.9-1.2)	6327 (5580-7234)	1.3 (1.2-1.5)	0.8392 (0.7803-0.8981)
Oceania	60 (43-76)	1.9 (1.4-2.3)	183 (135-236)	2.2 (1.6-2.8)	0.6785 (0.5818-0.7753)
South Asia	49608 (44763-54895)	7.9 (7.1-8.7)	152646 (131077-170703)	9.8 (8.5-10.9)	0.5662 (0.4527-0.6798)
Southeast Asia	9187 (8040-10245)	3.5 (3.1-3.9)	27647 (23814-31578)	4.2 (3.6-4.8)	0.4198 (0.3705-0.4691)
Southern Latin America	1361 (1247-1482)	2.9 (2.7-3.2)	2177 (1970-2383)	2.6 (2.3-2.8)	-0.1705 (-0.4026-0.0622)
Southern Sub-Saharan Africa	1137 (877-1347)	3.9 (3.0-4.7)	2506 (2208-2801)	4.1 (3.6-4.5)	-0.0300 (-0.1748-0.1151)
Tropical Latin America	3438 (3267-3601)	3.6 (3.4-3.8)	9814 (9161-10427)	3.8 (3.5-4.0)	0.0724 (-0.0283-0.1733)
Western Europe	31991(30658-33257)	6.1 (5.8-6.3)	46069 (42748-48717)	5.6 (5.3-5.9)	-0.1889 (-0.2550--0.1228)
Western Sub-Saharan Africa	940 (761-1099)	1.0 (0.8-1.2)	2819 (2216-3442)	1.3 (1.1-1.6)	0.8038 (0.7445-0.8632)
Female	Incidence				
Global	55690 (52276-59041)	2.6 (2.4-2.7)	148660 (135703-160404)	3.3 (3.0-3.5)	0.6907 (0.6121-0.7695)
High-middle SDI	8939 (8357-9492)	1.6 (1.5-1.7)	22516 (20180-24622)	2.2 (1.9-2.4)	0.9264 (0.8395-1.0134)
High SDI	17964 (16803-18604)	2.9 (2.8-3.0)	36045 (31689-38609)	3.4 (3.1-3.6)	0.5455 (0.4696-0.6214)
Low-middle SDI	13621 (11888-15392)	4.2 (3.6-4.7)	43158 (37580-49407)	5.5 (4.8-6.3)	0.8052 (0.6984-0.9121)
Low SDI	3756 (3114-4364)	3.1 (2.6-3.6)	10593 (8941-12392)	3.8 (3.2-4.4)	0.4361 (0.3124-0.5598)
Middle SDI	11359 (10459-12230)	2.1 (1.9-2.2)	36244 (32427-40239)	2.6 (2.3-2.9)	0.5103 (0.3879-0.633)
Andean Latin America	133 (115-151)	1.2 (1.1-1.4)	503 (398-630)	1.6 (1.3-2.0)	0.8642 (0.6851-1.0437)
Australasia	530 (480-568)	4.2 (3.8-4.5)	1194 (1042-1325)	4.5 (4.0-5.0)	0.2746 (0.0343-0.5155)
Caribbean	324 (304-346)	2.4 (2.2-2.6)	598 (523-679)	2.1 (1.8-2.4)	-0.2869 (-0.4151--0.1584)
Central Asia	402 (367-443)	1.4 (1.3-1.6)	803 (695-912)	1.7 (1.5-1.9)	0.5910 (0.2850-0.8980)
Central Europe	1399 (1332-1463)	1.7 (1.6-1.8)	3056 (2779-3306)	2.6 (2.4-2.8)	1.4885 (1.4116-1.5654)
Central Latin America	646 (619-669)	1.5 (1.4-1.5)	1999 (1747-2258)	1.5 (1.3-1.7)	-0.0801 (-0.2099-0.0499)
Central Sub-Saharan Africa	193 (143-251)	1.6 (1.1-2.1)	546 (372-794)	1.7 (1.2-2.6)	0.2725 (0.1444-0.4008)
East Asia	5591 (4605-6680)	1.2 (1.0-1.5)	15657 (12336-19421)	1.4 (1.1-1.8)	0.3401 (0.1486-0.532)
Eastern Europe	2917 (2720-3084)	1.7 (1.6-1.8)	5840 (5232-6534)	3.0 (2.7-3.4)	1.7539 (1.5160-1.9923)
Eastern Sub-Saharan Africa	969 (836-1155)	2.4 (2.1-2.8)	2449 (2005-2989)	2.5 (2.1-3.0)	-0.0108 (-0.1210-0.0996)
High-income Asia Pacific	2176 (2003-2313)	2.0 (1.8-2.1)	7229 (5701-8207)	2.9 (2.5-3.2)	1.2882 (0.9727-1.6046)
High-income North America	8515 (7922-8845)	4.4 (4.1-4.5)	12620 (11417-13369)	3.8 (3.5-4.0)	-0.5690 (-0.6747--0.4631)
North Africa and Middle East	735 (640-845)	0.9 (0.7-1.0)	2735 (2396-3147)	1.2 (1.0-1.3)	1.2730 (1.1681-1.3781)
Oceania	22 (16-29)	1.4 (1.1-1.9)	65 (49-86)	1.7 (1.3-2.2)	0.5348 (0.4427-0.6270)
South Asia	17783 (15343-20148)	5.9 (5.1-6.7)	60219 (52360-69313)	7.7 (6.7-8.8)	0.6734 (0.5205-0.8265)
Southeast Asia	3971 (3380-4511)	2.9 (2.5-3.3)	11125 (9383-13161)	3.3 (2.8-3.9)	0.0901 (0.0050-0.1753)
Southern Latin America	332 (311-351)	1.3 (1.2-1.4)	760 (691-824)	1.6 (1.4-1.7)	1.0493 (0.8027-1.2965)
Southern Sub-Saharan Africa	330 (271-395)	2.0 (1.7-2.5)	848 (749-961)	2.5 (2.2-2.8)	0.8037 (0.6012-1.0065)
Tropical Latin America	819 (770-855)	1.7 (1.6-1.8)	2777 (2534-2943)	2.0 (1.8-2.1)	0.3208 (0.1954-0.4464)
Western Europe	7366 (6906-7654)	2.4 (2.3-2.5)	15947 (14137-17124)	3.5 (3.2-3.7)	1.4558 (1.3099-1.6020)
Western Sub-Saharan Africa	528 (418-631)	1.2 (0.9-1.4)	1681 (1300-2142)	1.5 (1.2-1.9)	0.8315 (0.7870-0.8761)
Male	Incidence				
Global	118387 (112776-124445)	6.2 (5.9-6.5)	272917 (245321-296015)	6.7 (6.0-7.2)	0.2294 (0.1648-0.2941)
High-middle SDI	27745 (26682-28937)	6.1 (5.8-6.3)	52865 (47657-58782)	5.8 (5.3-6.5)	-0.2089 (-0.3050--0.1127)
High SDI	40238 (38980-41402)	8.5 (8.2-8.7)	68826 (65517-71809)	7.7 (7.3-8.0)	-0.2540 (-0.3085--0.1995)
Low-middle SDI	21899 (18789-25089)	6.6 (5.6-7.5)	58881 (48621-67462)	8.0 (6.7-9.1)	0.5842 (0.5139-0.6546)
Low SDI	5744 (4716-6786)	4.7 (3.9-5.6)	14496 (11581-17286)	5.4 (4.3-6.4)	0.2913 (0.2007-0.3819)
Middle SDI	22621 (20849-24615)	4.2 (3.9-4.6)	77613 (66548-87891)	5.9 (5.1-6.7)	1.1157 (1.0162-1.2152)
Andean Latin America	150 (128-176)	1.5 (1.2-1.7)	442 (348-558)	1.6 (1.2-2.0)	0.3509 (0.1690-0.5330)
Australasia	1161 (1025-1307)	10.8 (9.6-12.2)	2274 (1965-2614)	9.7 (8.4-11.1)	-0.3813 (-0.5588--0.2035)
Caribbean	744 (685-809)	5.9 (5.5-6.4)	1513 (1293-1748)	6.0 (5.1-6.9)	0.2477 (0.1114-0.3842)
Central Asia	861 (801-938)	4.2 (3.9-4.6)	1254 (1091-1449)	3.3 (2.9-3.8)	-0.6827 (-0.9341--0.4307)
Central Europe	4964 (4724-5251)	7.5 (7.1-7.9)	8348 (7598-9112)	9.1 (8.3-10.0)	0.5412 (0.4101-0.6726)
Central Latin America	883 (847-921)	2.2 (2.1-2.3)	2443 (2138-2752)	2.1 (1.9-2.4)	-0.3947 (-0.5126--0.2767)
Central Sub-Saharan Africa	270 (199-399)	2.5 (1.9-3.5)	761 (572-993)	2.8 (2.1-3.6)	0.3741 (0.2004-0.5482)
East Asia	10429 (8674-12391)	2.4 (2.0-2.8)	47856 (37470-60188)	4.6 (3.6-5.7)	2.4966 (2.3051-2.6885)
Eastern Europe	10284 (9842-11040)	9.3 (8.9-9.9)	15198 (13344-17068)	11.0 (9.7-12.4)	0.2457 (0.0599-0.4319)
Eastern Sub-Saharan Africa	1613 (1355-1864)	4.1 (3.4-4.7)	3887 (2987-4749)	4.4 (3.5-5.3)	0.1274 (0.0665-0.1882)
High-income Asia Pacific	3741 (3584-3897)	4.2 (4.0-4.4)	10959 (9948-11869)	5.7 (5.2-6.2)	0.8593 (0.5371-1.1826)
High-income North America	15610 (15137-16027)	10.6 (10.2-10.8)	23944 (22612-25016)	8.2 (7.8-8.6)	-0.7420 (-0.8528--0.6311)
North Africa and Middle East	1101 (929-1290)	1.2 (1.0-1.5)	3591 (3103-4115)	1.5 (1.3-1.7)	0.5225 (0.4813-0.5638)
Oceania	38 (26-49)	2.3 (1.6-2.9)	118 (84-153)	2.7 (1.9-3.5)	0.7578 (0.6574-0.8584)
South Asia	31824 (27673-36043)	9.7 (8.3-11.0)	92426 (73789-107477)	12.0 (9.6-13.9)	0.5758 (0.4880-0.6637)
Southeast Asia	5216 (4461-5961)	4.2 (3.6-4.7)	16522 (13976-19047)	5.2 (4.4-6.0)	0.6546 (0.6157-0.6936)
Southern Latin America	1028 (919-1148)	4.8 (4.3-5.4)	1416 (1235-1613)	3.7 (3.2-4.2)	-0.6689 (-0.9129--0.4242)
Southern Sub-Saharan Africa	806 (586-996)	6.3 (4.5-7.8)	1658 (1411-1863)	6.2 (5.3-6.9)	-0.3475 (-0.5148--0.1799)
Tropical Latin America	2618 (2473-2758)	5.7 (5.4-6.0)	7036 (6543-7597)	5.9 (5.4-6.3)	0.0427 (-0.0673-0.1529)
Western Europe	24624 (23573-25815)	10.4(10.0-10.9)	30121 (27818-32159)	7.9 (7.4-8.5)	-0.8810 (-0.9396--0.8224)
Western Sub-Saharan Africa	411 (326-502)	0.9 (0.7-1.0)	1138 (868-1421)	1.1 (0.8-1.3)	0.6852 (0.5952-0.7753)

**Table 2:** Changes in lip and oral cavity cancer Deaths by regions and sexes.

Locations	1990 Number (95% UI, cases)	1990 Age-standardized rate (95% UI, per 100,000)	2021 Number (95% UI, cases)	2021 Age-standardized rate (95% UI, per 100,000)	Estimated annual percentage change (95% CI, 100%)
Both sexes					
Global	97402 (92506-102361)	2.5 (2.3-2.6)	208379 (191288-224162)	2.4 (2.2-2.6)	-0.1142 (-0.1675--0.0608)
High-middle SDI	19478 (18676-20329)	2.0 (1.9-2.1)	31196 (28448-33893)	1.6 (1.5-1.7)	-0.8570 (-0.9267--0.7872)
High SDI	20812 (19950-21348)	1.9 (1.8-2.0)	31747 (29014-33462)	1.5 (1.4-1.6)	-0.7532 (-0.8076--0.6988)
Low-middle SDI	26672 (23882-29567)	4.3 (3.8-4.7)	67360 (59761-74529.)	4.7 (4.1-5.1)	0.1991 (0.1431-0.2551)
Low SDI	7278 (6331-8263)	3.2 (2.8-3.6)	17333 (14819-19852)	3.4 (2.9-3.9)	0.0747 (-0.0083-0.1578)
Middle SDI	23048 (21514-24578)	2.3 (2.1-2.4)	60565 (54822-66426)	2.3 (2.1-2.5)	-0.0579 (-0.1052--0.0106)
Andean Latin America	206 (179-235)	1.0 (0.9-1.2)	537 (426-658)	0.9 (0.7-1.1)	-0.2902 (-0.4251--0.1552)
Australasia	420 (391-448)	1.8 (1.7-1.9)	720 (643-793)	1.3 (1.2-1.5)	-0.9718 (-1.2489--0.6940)
Caribbean	664 (620-715)	2.6 (2.5-2.8)	1202 (1041-1376)	2.2 (1.9-2.5)	-0.3013 (-0.4092--0.1932)
Central Asia	894 (828-973)	1.9 (1.7-2.0)	1284 (1124-1460)	1.6 (1.4-1.8)	-0.6121 (-0.7923--0.4315)
Central Europe	4116 (3937-4305)	2.8 (2.7-3.0)	6001 (5520-6457)	2.9 (2.7-3.1)	0.014 (-0.0592-0.0873)
Central Latin America	1052 (1014-1084)	1.3 (1.3-1.4)	2553 (2271-2856)	1.0 (0.9-1.2)	-0.9621 (-1.0695--0.8546)
Central Sub-Saharan Africa	366 (285-474)	1.7 (1.4-2.1)	951 (721-1223)	1.8 (1.3-2.3)	0.1094 (0.0010-0.2179)
East Asia	10468 (8942-11942)	1.2 (1.1-1.4)	26513 (21531-32205)	1.2 (1.0-1.5)	0.0767 (-0.0391-0.1926)
Eastern Europe	7191 (6920-7572)	2.6 (2.5-2.7)	9009 (8172-9925)	2.7 (2.4-3.0)	-0.3604 (-0.5531--0.1673)
Eastern Sub-Saharan Africa	2016 (1768-2307)	2.7 (2.4-3.1)	4492 (3620-5418)	2.6 (2.1-3.1)	-0.198 (-0.2449--0.1511)
High-income Asia Pacific	1976 (1872-2048)	1.0 (0.9-1.0)	5917 (4977-6492)	1.1 (1.0-1.2)	0.0541 (-0.2924-0.4017)
High-income North America	6809 (6495-7009)	2.0 (1.9-2.0)	8855 (8160-9270)	1.3 (1.2-1.4)	-1.2256 (-1.3958--1.0551)
North Africa and Middle East	1201 (1048-1361)	0.7 (0.6-0.8)	3089 (2728-3518)	0.7 (0.6-0.8)	-0.1222 (-0.1652--0.0791)
Oceania	40 (29-51)	1.4 (1.0-1.7)	120 (87-155)	1.6 (1.2-2.0)	0.6374 (0.5483-0.7265)
South Asia	36681 (32922-40660)	6.2 (5.5-6.9)	97154 (84355-108404)	6.5 (5.7-7.)	0.0274 (-0.0546-0.1095)
Southeast Asia	6096 (5308-6820)	2.5 (2.2-2.8)	15400 (13307-17498)	2.5 (2.2-2.8)	-0.1751 (-0.2264--0.1238)
Southern Latin America	788 (723-860)	1.7 (1.6-1.9)	1074 (975-1170)	1.2 (1.1-1.3)	-0.7292 (-0.9522--0.5057)
Southern Sub-Saharan Africa	771 (587-923)	2.8 (2.1-3.4)	1605 (1417-1788)	2.7 (2.4-3.0)	-0.2362 (-0.4675--0.0043)
Tropical Latin America	2310 (2193-2425)	2.5 (2.4-2.7)	5714 (5312-6066)	2.2 (2.1-2.3)	-0.4524 (-0.5593--0.3454)
Western Europe	12607 (12109-13022)	2.3 (2.2-2.4)	14227 (12881-15084)	1.5 (1.4-1.6)	-1.2628 (-1.3406--1.1850)
Western Sub-Saharan Africa	719 (586-841)	0.8 (0.7-2.0)	1955 (1557-2358)	1.0 (0.8-1.2)	0.5845 (0.5317-0.6373)
Female					
Global	30412 (28095-32661)	1.4 (1.3-1.6)	71488 (64699-78442)	1.6 (1.4-1.7)	0.1424 (0.0649-0.2199)
High-middle SDI	4764 (4441-5084)	0.9 (0.8-0.9)	8753 (7833-9480)	0.8 (0.7-0.9)	-0.4395 (-0.5133--0.3657)
High SDI	6154 (5666-6398)	1.0 (0.9-1.0)	10845 (9172-11834)	0.9 (0.8-0.9)	-0.3224 (-0.3966--0.2481)
Low-middle SDI	9638 (8334-10938)	3.2 (2.7-3.6)	26857 (23443-30604)	3.6 (3.2-4.1)	0.3532 (0.2677-0.4387)
Low SDI	2715 (2268-3167)	2.4 (2.0-2.8)	6875 (5882-8044)	2.7 (2.3-3.1)	0.1918 (0.0865-0.2972)
Middle SDI	7112 (6553-7662)	1.4 (1.3-1.5)	18106 (16077-20180)	1.3 (1.2-1.5)	-0.4032 (-0.4804--0.3259)
Andean Latin America	92 (81-105)	0.9 (0.8-1.0)	271 (216-337)	0.9 (0.7-1.1)	-0.1211 (-0.2697-0.0278)
Australasia	135 (124-144)	1.0 (0.9-1.1)	251 (210-279)	0.8 (0.7-0.9)	-0.6814 (-0.9861--0.3758)
Caribbean	191 (179-204)	1.5 (1.4-1.6)	335 (294-384)	1.1 (1.0-1.3)	-0.6148 (-0.7389--0.4906)
Central Asia	268 (239-299)	1.0 (0.9-1.1)	457 (399-517)	1.0 (0.9-1.1)	0.0705 (-0.1610-0.3024)
Central Europe	839 (801-880)	1.0 (1.0-1.1)	1486 (1338-1604)	1.2 (1.1-1.3)	0.5257 (0.4347-0.6167)
Central Latin America	423 (405-439)	1.1 (1.0-1.1)	1083 (948-1212)	0.8 (0.7-0.9)	-0.8609 (-1.0029--0.7187)
Central Sub-Saharan Africa	145 (108-189)	1.3 (1.0-1.7)	379 (254-551)	1.3 (0.9-2.0)	0.0417 (-0.0280-0.1115)
East Asia	3333 (2755-3961)	0.8 (0.7-0.9)	5742 (4554-7078)	0.5 (0.4-0.6)	-1.7080 (-1.8764--1.5394)
Eastern Europe	1541 (1450-1615)	0.9 (0.8-0.9)	2338 (2096-2604)	1.1 (1.0-1.2)	0.4123 (0.2722-0.5525)
Eastern Sub-Saharan Africa	714 (616-854)	1.9 (1.2-2.3)	1622 (1340-1960)	1.8 (1.5-2.2)	-0.2713 (-0.3419--0.2007)
High-income Asia Pacific	691 (629-728)	0.6 (0.6-0.7)	2670 (1997-3098)	0.8 (0.6-0.9)	0.3256 (-0.0301-0.6825)
High-income North America	2386 (2174-2497)	1.2 (1.1-1.2)	3011 (2628-3222)	0.8 (0.7-0.9)	-1.2177 (-1.3831--1.0520)
North Africa and Middle East	453 (390-517)	0.6 (0.5-0.7)	1234 (1085-1402)	0.6 (0.5-0.7)	0.2767 (0.1808-0.3726)
Oceania	12 (9-16)	1.0 (0.7-1.2)	37 (28-49)	1.1 (0.8-1.4)	0.5564 (0.4855-0.6273)
South Asia	12410 (10628-14112)	4.5 (3.7-5.1)	36201 (31470-41708)	4.8 (4.2-5.6)	0.1190 (-0.0025-0.2407)
Southeast Asia	2393 (2055-2724)	1.9 (1.7-2.2)	5768 (4856-6823)	1.8 (1.5-2.1)	-0.4598 (-0.5641--0.3554)
Southern Latin America	197 (183-207)	0.8 (0.7-0.8)	382 (343-413)	0.7 (0.7-0.8)	0.3250 (0.0915-0.5591)
Southern Sub-Saharan Africa	210 (171-255)	1.4 (1.1-1.7)	518 (460-584)	1.6 (1.4-1.8)	0.5812 (0.3059-0.8574)
Tropical Latin America	539 (503-565)	1.2 (1.1-1.3)	1570 (1410-1677)	1.1 (1.0-1.2)	-0.3237 (-0.4545--0.1928)
Western Europe	3036 (2804-3161)	0.9 (0.8-0.9)	4997 (4261-5434)	0.9 (0.8-1.0)	0.1425 (0.0588-0.2262)
Western Sub-Saharan Africa	393 (311-471)	0.9 (0.7-1.1)	1127 (890-1425)	1.1 (0.9-1.4)	0.6491 (0.5864-0.7118)
Male					
Global	66990 (62782-71608)	3.6 (3.4-3.9)	136890 (120655-149372)	3.4 (3.0-3.7)	-0.2789 (-0.3244--0.2334)
High-middle SDI	14714 (14079-15385)	3.4 (3.2-3.5)	22443 (20142-24822)	2.5 (2.3-2.8)	-1.0733 (-1.1405--1.006)
High SDI	14657 (14191-15051)	3.2 (3.0-3.2)	20902 (19819-21889)	2.3 (2.1-2.4)	-1.0570 (-1.1120--1.0020)
Low-middle SDI	17034 (14536-19575)	5.4 (4.6-6.2)	40502 (33644-46284)	5.8 (4.8-6.6)	0.1638 (0.1226-0.2050)
Low SDI	4562 (3778-5393)	3.9 (3.3-4.7)	10457 (8399-12440)	4.1 (3.3-4.9)	0.0369 (-0.0350-0.1088)
Middle SDI	15935 (14660-17390)	3.2 (2.9-3.5)	42458 (36636-47416)	3.3 (2.9-3.7)	0.1388 (0.0926-0.1849)
Andean Latin America	113 (96-133)	1.2 (1.0-1.4)	266 (211-334)	1.0 (0.8-1.2)	-0.4436 (-0.5954--0.2915)
Australasia	284 (257-313)	2.7 (2.5-3.0)	468 (408-529)	1.9 (1.7-2.1)	-1.1781 (-1.4448--0.9107)
Caribbean	473 (437-518)	3.9 (3.6-4.2)	866 (737-1007)	3.4 (2.9-4.0)	-0.1469 (-0.2787--0.0149)
Central Asia	625 (582-683)	3.2 (3.0-3.5)	827 (722-952)	2.3 (2.0-2.6)	-1.0245 (-1.2088--0.8399)
Central Europe	3277 (3119-3450)	5.1 (4.8-5.3)	4515 (4144-4895)	4.9 (4.5-5.4)	-0.1864 (-0.2846--0.0881)
Central Latin America	628 (602-653)	1.6 (1.6-1.7)	1469 (1286-1657)	1.3 (1.1-1.5)	-0.9860 (-1.099--0.8729)
Central Sub-Saharan Africa	220 (162-322)	2.1 (1.6-3.0)	572 (429-749)	2.3 (1.8-2.9)	0.2063 (0.0856-0.3272)
East Asia	7135 (5907-8495)	1.8 (1.5-2.1)	20770 (16201-26057)	2.1 (1.6-2.6)	0.7324 (0.5876-0.8775)
Eastern Europe	5649 (5405-6018)	5.3 (5.1-5.7)	6670 (5872-7520)	4.8 (4.3-5.5)	-0.7124 (-0.9048--0.5197)
Eastern Sub-Saharan Africa	1302 (1103-1522)	3.5 (2.9-4.0)	2869 (2234-3500)	3.5 (2.8-4.2)	-0.0706 (-0.1045--0.0366)
High-income Asia Pacific	1285 (1241.4-1327)	1.5 (1.4-1.5)	3246 (2971-3434)	1.6 (1.4-1.6)	-0.2271 (-0.5713-0.1183)
High-income North America	4423 (4283-4542)	3.0 (2.9-3.1)	5844 (5475-6103)	2.0 (1.8-2.1)	-1.3253 (-1.5100--1.1404)
North Africa and Middle East	748 (625-881)	0.9 (0.8-1.1)	1855 (1612-2123)	0.8 (0.7-0.9)	-0.3956 (-0.4495--0.3417)
Oceania	27 (19-36)	1.8 (1.2-2.3)	82 (58-108)	2.0 (1.5-2.7)	0.6716 (0.5714-0.7719)
South Asia	24271 (21049-27547)	7.8 (6.7-8.9)	60952 (49383-70777)	8.3 (6.7-9.5)	0.0639 (0.0022-0.1255)
Southeast Asia	3702 (3167-4226)	3.2 (2.7-3.6)	9632 (8243-11109)	3.2 (2.8-3.7)	0.0123 (-0.0128-0.0374)
Southern Latin America	591 (528-661)	2.9 (2.6-3.2)	691 (609-782)	1.8 (1.6-2.0)	-1.1603 (-1.3943--0.9256)
Southern Sub-Saharan Africa	560 (405-694)	4.6 (3.3-5.7)	1086 (930-1218)	4.3 (3.7-4.8)	-0.5300 (-0.776--0.2834)
Tropical Latin America	1771 (1675-1867)	4.1 (3.8-4.3)	4143 (3858-4452)	3.5 (3.3-3.8)	-0.4345 (-0.5494--0.3195)
Western Europe	9570 (9164-9940)	4.1 (3.9-4.2)	9230 (8525-9803)	2.3 (2.1-2.4)	-1.8678 (-1.9557--1.7799)
Western Sub-Saharan Africa	325 (257-396)	0.7 (0.6-0.9)	828 (642-1033)	0.8 (0.7-1.0)	0.4618 (0.3889-0.5348)

**Table 3:** Changes in lip and oral cavity cancer Prevalence by regions and sexes.

Locations	1990 Number (95% UI, cases)	1990 Age-standardized rate (95% UI, per 100,000)	2021 Number (95% UI, cases)	2021 Age-standardized rate (95% UI, per 100,000)	Estimated annual percentage change (95% CI, 100%)
Both sexes					
Global	587479 (568848-605472)	13.9 (13.4-14.3)	1538007 (1435013-1633910)	17.7 (16.5-18.8)	0.8167 (0.7644-0.869)
High-middle SDI	119288 (114980-123539)	11.5 (11.1-11.9)	291257 (266249-315946)	15.1 (13.9-16.4)	0.9095 (0.8257-0.9934)
High SDI	265628 (256944-271581)	25.1 (24.4-25.7)	497975 (472450-517411)	27.3 (26.1-28.3)	0.3903 (0.3151-0.4656)
Low-middle SDI	85381 (77410-94379)	11.7 (10.6-12.9)	287203 (251575-320280)	17.7 (15.6-19.6)	1.2932 (1.1636-1.4230)
Low SDI	21900 (18812-25057)	8.0 (6.9-9.1)	67580 (57110-79277)	10.8 (9.2-12.5)	0.8007 (0.6576-0.944)
Middle SDI	94680 (88988-100398)	7.8 (7.3-8.3)	392726 (354064-434502)	13.9 (12.6-15.4)	1.8834 (1.7527-2.0142)
Andean Latin America	718 (625-813)	3.1 (2.7-3.5)	3158 (2493-3920)	5.1 (4.0-6.4)	1.8574 (1.6934-2.0215)
Australasia	8540 (7867-9235)	37.2 (34.3-40.3)	19360 (17292-21470)	40.6 (36.6-44.8)	0.3218 (0.1694-0.4743)
Caribbean	3164 (2979-3356)	11.7 (11.0-12.4)	7054 (6075-8094)	13.1 (11.3-15.1)	0.6574 (0.5381-0.7768)
Central Asia	3584 (3358-3873)	7.0 (6.6-7.6)	6703 (5856-7588)	7.4 (6.5-8.3)	0.2509 (-0.0929-0.5958)
Central Europe	19525 (18539-20432)	13.2 (12.5-13.8)	42578 (38925-46197)	21.8 (19.9-23.7)	1.5905 (1.4366-1.7446)
Central Latin America	4135 (3997-4264)	4.4 (4.3-4.5)	14706 (13022-16593)	5.7 (5.1-6.4)	0.7209 (0.635-0.8069)
Central Sub-Saharan Africa	1043 (794-1356)	3.8 (3.0-4.9)	3470 (2626-4487)	5.0 (3.8-6.4)	0.8411 (0.593-1.0898)
East Asia	45700 (39379-51927)	4.6 (3.9-5.2)	250064 (206079-300702)	11.4 (9.5-13.7)	3.3177 (3.1823-3.4533)
Eastern Europe	32742 (31288-35020)	11.8 (11.3-12.7)	61763 (55837-67693)	19.6 (17.8-21.5)	1.4589 (1.2156-1.7027)
Eastern Sub-Saharan Africa	5856 (5113-6673)	6.5 (5.7-7.3)	17283 (13570-21137)	8.0 (6.4-9.6)	0.5903 (0.4352-0.7457)
High-income Asia Pacific	20976 (20119-21796)	10.2 (9.8-10.6)	60153 (54034-65073)	16.8 (15.3-18.0)	1.7110 (1.3867-2.0363)
High-income North America	123452 (119575-126380)	37.4 (36.3-38.2)	197418 (187715-205139)	32.6 (31.1-33.8)	-0.3975 (-0.4695--0.3255)
North Africa and Middle East	5393 (4785-6218)	2.7 (2.4-3.1)	24545 (21526-28272)	4.7 (4.1-5.4)	1.8601 (1.7726-1.9476)
Oceania	178 (129-226)	4.7 (3.4-5.9)	562 (420-721)	5.8 (4.4-7.4)	0.8092 (0.6957-0.9228)
South Asia	122433 (111426-135083)	17.4 (15.8-19.1)	447882 (382112-503421)	26.9 (23.1-30.2)	1.3426 (1.1874-1.498)
Southeast Asia	27164 (23868-30398)	9.2 (8.1-10.3)	98530 (84512-113952)	13.9 (12.0-16.1)	1.1642 (1.0764-1.2521)
Southern Latin America	4177 (3837-4538)	8.9 (8.2-9.6)	7874 (7116-8644)	9.5 (8.6-10.4)	0.4896 (0.2321-0.7476)
Southern Sub-Saharan Africa	3092 (2425-3626)	9.8 (7.6-11.6)	7127 (6250-8030)	10.7 (9.4-12.0)	0.1425 (0.0827-0.2023)
Tropical Latin America	9497 (9032-9936)	9.2 (8.7-9.6)	32002 (30163-33901)	12.1 (11.4-12.8)	0.7947 (0.7004-0.889)
Western Europe	143929 (138625-149532)	28.2 (27.2-29.3)	228216 (213878-240059)	29.7 (28.1-31.1)	0.2621 (0.1562-0.3681)
Western Sub-Saharan Africa	2170 (1735-2552)	2.1 (1.7-2.5)	7548 (5755-9413)	3.1 (2.4-3.7)	1.1718 (1.0441-1.2997)
Female					
Global	850043 (783392-917046)	38.6 (35.6-41.7)	1846853 (1681594-2028079)	41 (37.6-45.4)	0.0802 (-0.0104-0.1709)
High-middle SDI	31653 (29797-33536)	5.6 (5.3-6.0)	100068 (90014-109916)	9.9 (9.0-10.9)	1.9178 (1.8475-1.9881)
High SDI	82592 (78596-85170)	14.0 (13.4-14.4)	171808 (156304-181701)	17.8 (16.6-18.7)	0.9036 (0.8214-0.9858)
Low-middle SDI	35062 (31040-39501)	9.7 (8.5-10.9)	129778 (112482-149017)	15.5 (13.5-17.8)	1.4644 (1.3225-1.6065)
Low SDI	9284 (7751-10839)	6.8 (5.6-7.9)	30791 (25762-36369)	9.6 (8.1-11.2)	0.9086 (0.7521-1.0653)
Middle SDI	34878 (32314-37569)	5.7 (5.3-6.2)	136584 (121840-151053)	9.5 (8.5-10.5)	1.4919 (1.3432-1.6409)
Andean Latin America	2542 (2217-2889)	21.9 (19.0-24.8)	6535 (5189-8182)	20.6 (16.4-25.8)	-0.2715 (-0.4313--0.1115)
Australasia	2938 (2730-3108)	23.8 (22.1-25.2)	4647 (4067-5090)	17.6 (15.8-19.2)	-0.8876 (-1.1778--0.5966)
Caribbean	4664 (4367-5012)	33.7 (31.7-36.2)	7448 (6474-8626)	26.4 (22.9-30.6)	-0.6179 (-0.7371--0.4985)
Central Asia	7278 (6745-7932)	25.7 (23.8-28.0)	12915 (11199-14721)	26.7 (23.1-30.4)	0.0732 (-0.1382-0.2850)
Central Europe	19903 (19067-20726)	24.8 (23.7-25.8)	31147 (28431-33614)	28.4 (26.0-30.7)	0.5564 (0.4848-0.6280)
Central Latin America	10850 (10496-11190)	23.3 (22.5-24.1)	25392 (22150-28696)	18.6 (16.2-21.0)	-0.7594 (-0.8946--0.6241)
Central Sub-Saharan Africa	4513 (3375-5930)	32.9 (24.5-43.0)	11394 (7726-16565)	33.1 (22.2-48.2)	-0.0141 (-0.0801-0.0520)
East Asia	96145 (79016-115852)	20.1 (16.6-24.1)	134049 (105253-166568)	12.1 (9.5-15.1)	-1.9484 (-2.1384--1.7581)
Eastern Europe	36926 (34799-38632)	22.2 (20.8-23.2)	56501 (50563-63297)	30.2 (27.0-34.0)	0.7648 (0.6266-0.9031)
Eastern Sub-Saharan Africa	22374 (19071-27054)	50.6 (43.5-60.6)	50101 (40567-62046)	47.3 (39.0-57.3)	-0.3689 (-0.4480--0.2898)
High-income Asia Pacific	15854 (14885-16541)	14.4 (13.6-15.1)	37837 (30494-42511)	15.6 (13.6-16.9)	-0.0364 (-0.3985-0.3269)
High-income North America	51162 (48262-52774)	27.4 (26.2-28.1)	59381 (54152-62478)	18.2 (17.0-19.1)	-1.3833 (-1.5798--1.1864)
North Africa and Middle East	13138 (11503-15420)	14.1 (12.3-16.2)	33743 (29408-38878)	13.9 (12.1-15.8)	0.0876 (0.0243-0.1508)
Oceania	419 (311-549)	24.2 (18.0-31.5)	1207 (889-1627)	27.5 (20.7-36.5)	0.5602 (0.4940-0.6265)
South Asia	396269 (344236-449100)	122.3 (105.3-139.3)	1054651 (907435-1219147)	129.5 (112.1-149.5)	0.0254 (-0.0913-0.1423)
Southeast Asia	64130 (55030-74030)	44.3 (37.9-50.6)	135852 (113406-161805)	38.6 (32.4-46.0)	-0.7102 (-0.8217--0.5986)
Southern Latin America	4621 (4374-4830)	18.3 (17.3-19.1)	7865 (7225-8415)	16.8 (15.5-18.0)	0.2112 (-0.0252-0.4481)
Southern Sub-Saharan Africa	6201 (5133-7363)	36.3 (29.8-43.3)	14606 (12885-16769)	41.0 (36.3-46.8)	0.6459 (0.3588-0.9338)
Tropical Latin America	13796 (13127-14392)	27.1 (25.6-28.4)	36671 (33848-38546)	26.0 (24.0-27.3)	-0.2974 (-0.4418--0.1528)
Western Europe	64889 (61781-66697)	22.0 (21.2-22.5)	91803 (82282-97602)	20.8 (19.2-21.8)	-0.0522 (-0.1273-0.0228)
Western Sub-Saharan Africa	11423 (8991-13685)	24.2 (19.1-29.0)	33098 (25282-42987)	27.5 (21.6-35.0)	0.4231 (0.3640-0.4823)
Male					
Global	393846 (379155-408550)	19.4 (18.7-20.2)	968572 (879563-1049185)	23.1 (21.0-25.0)	0.6198 (0.5648-0.6747)
High-middle SDI	87635 (84122-91341)	18.3 (17.6-19.1)	191189 (171647-213834)	20.9 (18.8-23.3)	0.4278 (0.3390-0.5168)
High SDI	183036 (177656-188375)	37.8 (36.7-38.9)	326167 (312705-339380)	37.3 (35.8-38.8)	0.0827 (0.0102-0.1554)
Low-middle SDI	50319 (43099-57511)	13.7 (11.7-15.6)	157424 (128460-181418)	19.9 (16.4-22.9)	1.2015 (1.0773-1.3257)
Low SDI	12616 (10278-14752)	9.2 (7.5-10.8)	36789 (28828-44267)	12.0 (9.5-14.5)	0.7405 (0.6040-0.8771)
Middle SDI	59802 (55137-64875)	9.9 (9.2-10.8)	256142 (218360-292906)	18.6 (15.9-21.2)	2.1410 (2.0038-2.2784)
Andean Latin America	361 (309-421)	3.2 (2.7-3.8)	1377 (1074-1738)	4.6 (3.6-5.8)	1.4741 (1.2825-1.6661)
Australasia	5872 (5197-6576)	53.9 (47.7-60.4)	12754 (10966-14724)	55.8 (47.8-64.3)	0.1295 (-0.0086-0.2678)
Caribbean	2148 (1983-2328)	16.5 (15.2-17.9)	4996 (4190-5847)	19.4 (16.4-22.7)	0.8384 (0.6923-0.9848)
Central Asia	2313 (2151-2509)	10.5 (9.7-11.4)	3682 (3210-4189)	9.0 (7.9-10.2)	-0.3772 (-0.7234--0.0297)
Central Europe	14912 (14116-15742)	21.8 (20.6-23.0)	30292 (27350-33137)	33.5 (30.2-36.7)	1.2858 (1.1054-1.4665)
Central Latin America	2290 (2191-2391)	5.1 (4.9-5.3)	7601 (6616-8629)	6.4 (5.5-7.2)	0.4660 (0.3594-0.5727)
Central Sub-Saharan Africa	575 (423-855)	4.6 (3.4-6.7)	1911 (1428-2531)	5.9 (4.5-7.6)	0.8111 (0.5499-1.0729)
East Asia	27391 (23012-32267)	5.5 (4.6-6.5)	178396 (141151-224934)	16.5 (13.1-20.7)	4.0502 (3.8578-4.2428)
Eastern Europe	22884 (21680-24987)	19.8 (18.8-21.6)	37248 (32467-42807)	27.2 (23.8-31.2)	0.8308 (0.6289-1.0332)
Eastern Sub-Saharan Africa	3467 (2914-3992)	7.8 (6.5-9.0)	9953 (7486-12230)	9.6 (7.4-11.8)	0.6070 (0.4693-0.7449)
High-income Asia Pacific	12843 (12294-13461)	13.6 (13.0-14.2)	36234 (32967-39522)	21.2 (19.4-23.1)	1.5211 (1.2095-1.8337)
High-income North America	79815 (77451-81891)	53.7 (52.1-55.1)	129298 (123344-134767)	45.0 (42.9-46.7)	-0.4733 (-0.5573--0.3892)
North Africa and Middle East	3092 (2641-3639)	3.1 (2.6-3.6)	13238 (11342-15160)	4.9 (4.3-5.6)	1.5289 (1.456-1.6018)
Oceania	101 (70-132)	5.2 (3.6-6.6)	332 (241-428)	6.7 (4.8-8.5)	0.9430 (0.8365-1.0495)
South Asia	75861 (66379-85548)	20.7 (18.0-23.3)	260373 (206422-305435)	31.5 (25.1-36.8)	1.3163 (1.1803-1.4526)
Southeast Asia	14271 (12192-16274)	10.1 (8.6-11.5)	56456 (46903-66522)	16.3 (13.6-19.1)	1.5000 (1.4200-1.5801)
Southern Latin America	3186 (2862-3534)	14.6 (13.1-16.1)	5142 (4487-5871)	13.4 (11.7-15.2)	-0.0895 (-0.3563-0.178)
Southern Sub-Saharan Africa	2114 (1566-2601)	15.1 (10.9-18.7)	4540 (3819-5155)	15.4 (13.1-17.3)	-0.2424 (-0.3463--0.1384)
Tropical Latin America	7204 (6818-7562)	14.4 (13.6-15.2)	22533 (21010-24318)	18.2 (17.0-19.7)	0.6784 (0.5758-0.7811)
Western Europe	112221 (107084-117705)	47.6 (45.4-49.9)	149335 (138322-159354)	41.0 (38.2-43.7)	-0.4608 (-0.5468--0.3746)
Western Sub-Saharan Africa	914 (717-1114)	1.7 (1.3-2.1)	2871 (2140-3617)	2.4 (1.8-3.0)	1.0508 (0.8960-1.2059)

**Table 4:** Changes in lip and oral cavity cancer DALYs by regions and sexes.

Locations	1990 Number (95% UI, cases)	1990 Age-standardized rate (95% UI, per 100,000)	2021 Number (95% UI, cases)	2021 Age-standardized rate (95% UI, per 100,000)	Estimated annual percentage change (95% CI, 100%)
Both sexes					
Global	2936205 (2793741-3092058)	69.3 (65.9-73.0)	5874069 (5326986-6347557)	67.7 (61.3-73.2)	-0.1650 (-0.2178--0.1121)
High-middle SDI	574936 (552159-599598)	55.7 (53.7-58.1)	834847 (764565-906224)	43.3 (39.7-47.0)	-1.0146 (-1.0917--0.9375)
High SDI	567133 (549836-582623)	54.1 (52.5-55.6)	739298 (698770-772933)	40.0 (38.2-41.7)	-0.9743 (-1.0132--0.9353)
Low-middle SDI	846886 (759575-940604)	119.9 (107.2-133.0)	2041323 (1773549-2273768)	128.4 (112.7-142.3)	0.1680 (0.1205-0.2155)
Low SDI	231414 (200547-263401)	87.8 (76.4-100.0)	542181 (458550-628171)	90.5 (77.3-104.0)	-0.0701 (-0.1498-0.0097)
Middle SDI	712596 (666093-761404)	61.0 (57.0-65.1)	1711811 (1539968-1877702)	61.1 (55.1-67.0)	-0.0680 (-0.1171--0.0190)
Andean Latin America	5839 (5065-6626)	25.9 (22.4-29.4)	13748 (10902-16887)	22.5 (17.927.7)	-0.4093 (-0.5524--0.2660)
Australasia	11122 (10354-11990)	48.7 (45.3-52.5)	16714 (15055-18427)	34.2 (31.1-37.6)	-1.1051 (-1.3580--0.8516)
Caribbean	17267 (16040-18899)	64.9 (60.3-70.9)	30477 (26005-35574)	56.6 (48.3-66.0)	-0.2204 (-0.3353--0.1053)
Central Asia	27173 (25372-29367)	53.5 (49.8-57.8)	38199 (33257-43855)	42.3 (36.9-48.3)	-0.8158 (-0.9764--0.6550)
Central Europe	122195 (116995-127869)	83.1 (79.6-86.8)	158444 (145874-170984)	82.6 (76.0-89.2)	-0.1461 (-0.2595--0.0326)
Central Latin America	28302 (27434-29080)	31.5 (30.4-32.4)	64214 (57048-72384)	25.1 (22.3-28.3)	-0.8990 (-1.0084--0.7895)
Central Sub-Saharan Africa	11519 (8890-15029)	44.4 (34.8-57.1)	30186 (22815-39392)	45.7 (34.7-58.8)	0.0779 (-0.0296-0.1856)
East Asia	315772 (267868-362039)	33.0 (28.1-37.7)	697683 (563154-853797)	31.9 (25.8-38.8)	0.0126 (-0.1046-0.1300)
Eastern Europe	218629 (210259-232556)	78.5 (75.6-83.7)	262247 (236250-289742)	81.7 (73.5-90.4)	-0.3580 (-0.5575--0.1582)
Eastern Sub-Saharan Africa	63267 (55142-72792)	72.6 (63.7-83.2)	143023 (112392-174329)	69.7 (56.0-84.1)	-0.2614 (-0.3148--0.2080)
High-income Asia Pacific	53404 (51574-55281)	26.2 (25.3-27.2)	108024 (96340-116039)	26.8 (24.6-28.5)	-0.2683 (-0.6213-0.0861)
High-income North America	176202 (170277-181404)	53.8 (52.1-55.3)	208746 (198194-218647)	34.2 (32.6-35.8)	-1.4237 (-1.5936--1.2536)
North Africa and Middle East	36036 (31673-41787)	19.0 (16.7-21.7)	89539 (78518-102823)	17.7 (15.6-20.2)	-0.2081 (-0.2384--0.1778)
Oceania	1348 (957-1753)	37.5 (27.1-47.9)	4005 (2875-5239)	43.3 (31.4-55.9)	0.6615 (0.5762-0.7469)
South Asia	1185409 (1073536-1311139)	174.5 (156.9-193.0)	2964855 (2541835-3330175)	182.3 (157.1-203.7)	0.0277 (-0.0383-0.0936)
Southeast Asia	178289 (154260-200931)	63.4 (55.1-71.0)	422230 (365827-481879)	61.0 (52.9-69.5)	-0.2512 (-0.3001--0.2023)
Southern Latin America	21908 (20020-23803)	46.9 (42.9-50.9)	26651 (24190-29219)	31.8 (28.8-34.8)	-0.9688 (-1.2010--0.7360)
Southern Sub-Saharan Africa	24312 (18793-28939)	79.7 (60.9-95.3)	49628 (43455-55690)	76.4 (67.3-85.4)	-0.2904 (-0.5237--0.0566)
Tropical Latin America	69446 (66329-72825)	68.8 (65.5-72.2)	157920 (148726-167280)	59.9 (56.3-63.4)	-0.5232 (-0.6517--0.3946)
Western Europe	346859 (333307-359027)	67.8 (65.3-70.1)	326627 (304681-344003)	41.5(39.1-43.5)	-1.6138 (-1.6700--1.5576)
Western Sub-Saharan Africa	21898 (17650-25674)	22.2 (18.0-25.9)	60898. (46881-74961)	25.9 (20.5-31.4)	0.4910 (0.4302-0.5519)
Female					
Global	870961 (803991-939990)	39.6 (36.5-42.7)	1904257 (1730273-2088526)	42.6 (38.6-46.8)	0.1010 (0.0111-0.1910)
High-middle SDI	123436 (115145-132211)	22.3 (20.8-23.9)	206999 (188577-223727)	20.3 (18.5-22.0)	-0.4500 (-0.5255--0.3744)
High SDI	142779 (135284-147269)	24.2 (23.0-24.9)	214391 (191581-228823)	20.9 (19.3-22.1)	-0.4516 (-0.5138--0.3895)
Low-middle SDI	307856 (271222-348678)	88.1 (76.8-100.1)	795539 (689891-916633)	97.9 (85.2-112.1)	0.2451 (0.1589-0.3314)
Low SDI	87151 (72756-101815)	66.9 (56.1-77.9)	213022 (180430-249562)	70.1 (60.0-82.0)	-0.0451 (-0.1539-0.0638)
Middle SDI	209045 (192476-226234)	36.0 (33.1-38.8)	473157 (420157-524951)	33.2 (29.6-36.8)	-0.4605 (-0.5659--0.3549)
Andean Latin America	2588 (2260-2937)	22.3 (19.4-25.3)	6728 (5336-8412)	21.3 (16.9-26.6)	-0.2346 (-0.3950--0.0739)
Australasia	3177 (2949-3366)	25.7 (23.9-27.2)	5203 (4593-5741)	19.7 (17.8-21.6)	-0.7636 (-1.0456--0.4808)
Caribbean	4781 (4478-5141)	34.6 (32.4-37.1)	7673 (6679-8906)	27.2 (23.6-31.6)	-0.6022 (-0.7215--0.4829)
Central Asia	7426 (6888-8102)	26.2 (24.3-28.7)	13232 (11450-15070)	27.3 (23.7-31.1)	0.0894 (-0.1241-0.3033)
Central Europe	20429 (19559-21296)	25.4 (24.3-26.5)	32397 (29701-34903)	29.5 (27.-31.8)	0.5971 (0.5256-0.6687)
Central Latin America	11075 (10703-11396)	23.8 (22.9-24.6)	26162 (22924-29589)	19.1 (16.8-21.6)	-0.7310 (-0.8658--0.5961)
Central Sub-Saharan Africa	4577 (3422-6022)	33.4 (24.9-43.7)	11585 (7862-16881)	33.7 (22.6-49.0)	-0.0070 (-0.0743-0.0604)
East Asia	98226 (80451-118410)	20.5 (16.9-24.6)	140871 (110107-175966)	12.8 (10.0-15.9)	-1.8521 (-2.0437--1.6601)
Eastern Europe	38036 (35839-39830)	22.8 (21.4-23.9)	58926 (52550-65875)	31.5 (28.0-35.3)	0.8105 (0.6702-0.9509)
Eastern Sub-Saharan Africa	22696 (19342-27439)	51.3(44.1-61.4)	50962 (41238-62955)	48.1 (39.6-58.2)	-0.3610 (-0.4408--0.2812)
High-income Asia Pacific	16706 (15707-17482)	15.2 (14.3-15.9)	40420 (32648-45492)	16.7 (14.7-18.2)	0.0532 (-0.3040-0.4116)
High-income North America	55050 (51826-57234)	29.4 (28.0-30.5)	65261 (59614-69022)	20.0 (18.6-21.1)	-1.3102 (-1.4983--1.1217)
North Africa and Middle East	13406 (11742-15698)	14.4 (12.6-16.6)	34866 (30475-40228)	14.3 (12.5-16.4)	0.1287 (0.0643-0.1932)
Oceania	427 (316-560)	24.7 (18.4-32.2)	1232 (909-1657)	28.0 (21.1-37.1)	0.5601 (0.4935-0.6268)
South Asia	402040 (348796-455621)	124.2 (106.9-140.9)	1075186 (924959-1242527)	132.0 (114.2-152.3)	0.0402 (-0.0770-0.1576)
Southeast Asia	65569 (56025-75728)	45.3 (38.7-51.9)	140137 (117294-166986)	39.8 (33.4-47.4)	-0.6828 (-0.7931--0.5725)
Southern Latin America	4741 (4481-4967)	18.7 (17.7-19.6)	8162 (7516-8728)	17.4 (16.2-18.6)	0.2468 (0.0104-0.4837)
Southern Sub-Saharan Africa	6317 (5249-7512)	37.0 (30.5-44.2)	14911 (13148-17131)	41.8 (37.0-47.8)	0.6515 (0.3666-0.9371)
Tropical Latin America	14080 (13388-14689)	27.7 (26.1-29.0)	37707 (34750-39638)	26.7 (24.7-28.1)	-0.2747 (-0.4181--0.1311)
Western Europe	68007 (64546-70339)	23.0 (22.1-23.7)	98948 (88447-105655)	22.5 (20.8-23.7)	0.0527 (-0.0235-0.1290)
Western Sub-Saharan Africa	11597 (9133-13887)	24.6 (19.4-29.4)	33680 (25776-43812)	28.0 (22.1-35.7)	0.4313 (0.3726-0.4900)
Male					
Global	66990 (62782-71608)	3.6 (3.4-3.9)	136890 (120655-149372)	3.4 (3.0-3.7)	-0.2789 (-0.3244--0.2334)
High-middle SDI	451499 (432553-473004)	94.5(90.5-98.9)	627847 (561697-694420)	68.6 (61.4-75.8)	-1.2245 (-1.3029--1.1460)
High SDI	424353 (411242-436619)	88.4 (85.6-90.9)	524906 (501600-549228)	60.3 (57.6-63.0)	-1.2373 (-1.2729--1.2017)
Low-middle SDI	539029 (462781-617695)	150.5 (128.7-172.3)	1245784 (1010032-1435569)	160.6 (131.8-184.1)	0.1783 (0.1489-0.2076)
Low SDI	144263 (119651-169530)	108.0 (89.5-127.2)	329159 (262644-392946)	111.5 (89.8-132.6)	-0.0490 (-0.1134-0.0154)
Middle SDI	503550 (461761-550025)	86.4 (79.5-94.2)	1238654 (1059945-1380995)	90.7 (77.9-101.2)	0.1396 (0.0968-0.1824)
Andean Latin America	113 (96-133)	1.2 (1.0-1.4)	266 (211-334)	1.0 (0.8-1.2)	-0.4436 (-0.5954--0.2915)
Australasia	284 (257-313)	2.7 (2.5-3.0)	468 (408-529)	1.9 (1.7-2.1)	-1.1781 (-1.4448--0.9107)
Caribbean	473 (437-518)	3.9 (3.6-4.2)	866 (737-1007)	3.4 (2.9-4.0)	-0.1469 (-0.2787--0.0149)
Central Asia	625 (582-683)	3.2 (3.0-3.5)	827 (722-952)	2.3 (2.0-2.6)	-1.0245 (-1.2088--0.8399)
Central Europe	3277 (3119-3450)	5.1 (4.8-5.3)	4515 (4144-4895)	4.9 (4.5-5.4)	-0.1864 (-0.2846--0.0881)
Central Latin America	628 (602-653)	1.6 (1.6-1.7)	1469 (1286-1657)	1.3 (1.1-1.5)	-0.9860 (-1.099--0.8729)
Central Sub-Saharan Africa	220 (162-322)	2.1 (1.6-3.0)	572 (429-749)	2.3 (1.8-2.9)	0.2063 (0.0856-0.3272)
East Asia	7135 (5907-8495)	1.8 (1.5-2.1)	20770 (16201-26057)	2.1 (1.6-2.6)	0.7324 (0.5876-0.8775)
Eastern Europe	5649 (5405-6018)	5.3 (5.1-5.7)	6670 (5872-7520)	4.8 (4.3-5.5)	-0.7124 (-0.9048--0.5197)
Eastern Sub-Saharan Africa	1302 (1103-1522)	3.5 (2.9-4.0)	2869 (2234-3500)	3.5 (2.8-4.2)	-0.0706 (-0.1045--0.0366)
High-income Asia Pacific	1285 (1241-1327)	1.5 (1.4-1.5)	3246 (2971-3434)	1.6 (1.4-1.6)	-0.2271 (-0.5713-0.1183)
High-income North America	4423 (4283-4542)	3.0 (2.9-3.1)	5844 (5475-6103)	2.0 (1.8-2.1)	-1.3253 (-1.5100--1.1404)
North Africa and Middle East	748 (625-881)	0.9 (0.8-1.1)	1855 (1612-2123)	0.8 (0.7-0.9)	-0.3956 (-0.4495--0.3417)
Oceania	27 (19-36)	1.8 (1.2-2.3)	82 (58-108)	2.0 (1.5-2.7)	0.6716 (0.5714-0.7719)
South Asia	24271 (21049-27547)	7.8 (6.7-8.9)	60952 (49383-70777)	8.3 (6.7-9.5)	0.0639 (0.0022-0.1255)
Southeast Asia	3702 (3167-4226)	3.2 (2.7-3.6)	9632 (8243-11109)	3.2 (2.8-3.7)	0.0123 (-0.0128-0.0374)
Southern Latin America	591 (528-661)	2.9 (2.6-3.2)	691 (609-782)	1.8 (1.6-2.0)	-1.1603 (-1.3943--0.9256)
Southern Sub-Saharan Africa	560 (405-694)	4.6 (3.3-5.7)	1086 (930-1218)	4.3 (3.7-4.8)	-0.5300 (-0.776--0.2834)
Tropical Latin America	1771 (1675-1867)	4.1 (3.8-4.3)	4143 (3858-4452)	3.5 (3.3-3.8)	-0.4345 (-0.5494--0.3195)
Western Europe	9570 (9164-9940)	4.1 (3.9-4.2)	9230 (8525-9803)	2.3 (2.1-2.4)	-1.8678 (-1.9557--1.7799)
Western Sub-Saharan Africa	325 (257-396)	0.7 (0.6-0.9)	828 (642-1033)	0.8 (0.7-1.0)	0.4618 (0.3889-0.5348)

**Table 5:** Changes in lip and oral cavity cancer YLLs by regions and sexes.

Locations	1990 Number (95% UI, cases)	1990 Age-standardized rate (95% UI, per 100,000)	2021 Number (95% UI, cases)	2021 Age-standardized rate (95% UI, per 100,000)	Estimated annual percentage change (95% CI, 100%)
Both sexes					
Global	2871519 (2733328-3026295)	67.7 (64.4-71.3)	5713196 (5183525-6171742)	65.9 (59.7-71.2)	-0.1832 (-0.2361--0.1302)
High-middle SDI	561447 (539389-585862)	54.4 (52.3-56.8)	804832 (738906-874361)	41.8 (38.3-45.3)	-1.0591 (-1.1369--0.9811)
High SDI	541955 (526962-553673)	51.8 (50.3-52.9)	693410 (656939-723548)	37.6 (36.0-39.1)	-1.0385 (-1.0781--0.9988)
Low-middle SDI	835581 (749392-929025)	118.2 (105.9-131.3)	2007285 (1746697-2238461)	126.2 (111.0-140.1)	0.1577 (0.1105-0.2048)
Low SDI	228405 (197877-260046)	86.6 (75.4-98.5)	533868 (451292-619265)	89.1 (76.0-102.3)	-0.0778 (-0.1575-0.0019)
Middle SDI	700957 (655320-747947)	60.0 (56.0-64.0)	1669323 (1502850-1830781)	59.6 (53.7-65.3)	-0.0960 (-0.1437--0.0482)
Andean Latin America	5742 (4973-6524)	25.4 (22.0-28.9)	13390 (10588-16439)	22.0 (17.4-26.9)	-0.4410 (-0.5831--0.2987)
Australasia	10356 (9674-11065)	45.4 (42.4-48.5)	15063 (13630-16529)	30.8 (28.0-33.8)	-1.214 (-1.4747--0.9527)
Caribbean	16889 (15691-18442)	63.5 (59.0-69.2)	29691 (25404-34636)	55.1 (47.2-64.3)	-0.2334 (-0.3486--0.1181)
Central Asia	26728 (25022-28953)	52.6 (49.0-56.9)	37426 (32549-42919)	41.4 (36.1-47.3)	-0.8312 (-0.9899--0.6723)
Central Europe	119896 (114789-125465)	81.6 (78.1-85.2)	153996.9393 (141726.3-166548.6)	80.4 (73.9-87.0)	-0.1752 (-0.2890--0.0613)
Central Latin America	27773 (26934-28544)	30.9 (29.9-31.8)	62534 (55474-70281)	24.5 (21.7-27.5)	-0.9226 (-1.0322--0.8129)
Central Sub-Saharan Africa	11365 (8773-14853)	43.7 (34.3-56.4)	29735 (22464-38796)	44.9 (34.1-57.8)	0.0723 (-0.0341-0.1789)
East Asia	310076 (263236-355895)	32.3 (27.6-36.9)	671860 (545021-826267)	30.7 (25.0-37.6)	-0.0524 (-0.1684-0.0637)
Eastern Europe	214353 (206458-227104)	77.7 (74.2-81.7)	254977 (229147-281922)	79.4 (71.4-87.7)	-0.3891 (-0.5900--0.1878)
Eastern Sub-Saharan Africa	62428 (54364-71641)	71.6 (62.7-81.9)	140859 (110404-171902)	68.6 (55.0-82.9)	-0.2676 (-0.3205--0.2146)
High-income Asia Pacific	51129 (49542-52724)	25.1 (24.3-25.9)	101422 (90072-109007)	25.1 (23.2-26.7)	-0.3531 (-0.7107-0.0059)
High-income North America	165135 (160216-168807)	50.5 (49.1-51.5)	191630 (181247-198978)	31.4 (30.0-32.6)	-1.4922 (-1.6690--1.3152)
North Africa and Middle East	35373 (31070-40913)	18.7 (16.4-21.3)	86975 (76178-99608)	17.2 (15.1-19.6)	-0.2432 (-0.2734--0.2130)
Oceania	1326 (941-1722)	36.9 (26.7-47.1)	3938 (2827-5161)	42.5 (30.9-55.0)	0.6608 (0.5756-0.7460)
South Asia	1169596 (1058465-1292454)	172.1 (154.7-190.7)	2913399 (2493288-3275522)	179.1 (154.1-200.3)	0.0152 (-0.0501-0.0806)
Southeast Asia	175044 (151362-197602)	62.2 (54.0-69.8)	411679 (356094-470571)	59.4 (51.6-67.7)	-0.2727 (-0.3221--0.2233)
Southern Latin America	21408 (19584-23345)	45.9 (42.0-49.9)	25787 (23468-28210)	30.7 (28.0-33.6)	-0.9987 (-1.2316--0.7652)
Southern Sub-Saharan Africa	23926 (18500-28431)	78.4 (60.0-93.8)	48750 (42680-54547)	75.0 (66.0-83.8)	-0.2968 (-0.5323--0.0608)
Tropical Latin America	68268 (65125-71560)	67.6 (64.3-70.9)	154326 (145322-163365)	58.5 (55.0-61.9)	-0.5410 (-0.6705--0.4113)
Western Europe	333109 (321050-343632)	65.3 (62.9-67.2)	305822 (286439-321093)	38.9 (36.8-40.7)	-1.7016 (-1.7595--1.6437)
Western Sub-Saharan Africa	21588 (17400-25352)	21.8 (17.7-25.6)	59926 (46200-73546)	25.5 (20.2-30.8)	0.4846 (0.4236-0.5455)
Female					
Global	850043 (783392-917046)	38.6 (35.6-41.7)	1846853 (1681594-2028079)	41.3 (37.6-45.4)	0.0802 (-0.0104-0.1709)
High-middle SDI	119984 (112275-129049)	21.7 (20.3-23.3)	197409 (179757-213284)	19.4 (17.7-20.9)	-0.5168 (-0.5923--0.4412)
High SDI	134989 (127968-138741)	22.9 (21.9-23.4)	198695 (177901-211359)	19.4 (17.9-20.4)	-0.5292 (-0.5935--0.4649)
Low-middle SDI	303425 (267186-343458)	86.8 (75.8-98.4)	780947 (678258-899360)	96.1 (83.7-109.9)	0.2330 (0.1468-0.3192)
Low SDI	85939 (71743-100350)	65.9 (55.2-76.8)	209454 (177431-245607)	68.9 (58.9-80.5)	-0.0544 (-0.1632-0.0544)
Middle SDI	205030 (189232-222063)	35.3 (32.5-38.1)	459239 (408646-510473)	32.2 (28.7-35.8)	-0.4936 (-0.5980--0.389)
Andean Latin America	2542 (2217-2889)	21.9 (19.0-24.8)	6535 (5189-8182)	20.6 (16.4-25.8)	-0.2715 (-0.4313--0.1115)
Australasia	2938 (2730-3108)	23.8 (22.1-25.2)	4647 (4067-5090)	17.6 (15.8-19.2)	-0.8876 (-1.1778--0.5966)
Caribbean	4664 (4367-5012)	33.7 (31.8-36.2)	7448 (6474-8626)	26.4 (22.8-30.6)	-0.6179 (-0.7371--0.4985)
Central Asia	7278 (6745-7932)	25.7 (23.8-28.0)	12915 (11199-14721)	26.7 (23.1-30.4)	0.0732 (-0.1382-0.2850)
Central Europe	19903 (19067-20726)	24.8 (23.7-25.8)	31147 (28431-33614)	28.4 (26.0-30.7)	0.5564 (0.4848-0.6280)
Central Latin America	10850 (10496-11190)	23.3 (22.5-24.1)	25392 (22150-28696)	18.6 (16.2-21.0)	-0.7594 (-0.8946--0.6241)
Central Sub-Saharan Africa	4513 (3375-5930)	32.9 (24.5-43.0)	11394 (7726-16565)	33.1 (22.2-48.2)	-0.0141 (-0.0801-0.0520)
East Asia	96145 (79016-115852)	20.1 (16.6-24.1)	134049 (105253-166568)	12.1 (9.5-15.1)	-1.9484 (-2.1384--1.7581)
Eastern Europe	36926 (34799-38632)	22.2 (20.8-23.2)	56501 (50563-63297)	30.2 (27.0-34.0)	0.7648 (0.6266-0.9031)
Eastern Sub-Saharan Africa	22374 (19071-27054)	50.6 (43.5-60.6)	50101 (40567-62046)	47.3 (39.0-57.3)	-0.3689 (-0.448--0.2898)
High-income Asia Pacific	850043 (783392-917046)	38.6 (35.6-41.7)	1846853 (1681594-2028079)	41.3 (37.6-45.4)	0.0802 (-0.0104-0.1709)
High-income North America	15854 (14885-16541)	14.4 (13.6-15.1)	37837 (30494-42511)	15.6 (13.6-16.9)	-0.0364 (-0.3985-0.3269)
North Africa and Middle East	51162 (48262-52774)	27.4 (26.2-28.1)	59381 (54152-62478)	18.2 (17.0-19.1)	-1.3833 (-1.5798--1.1864)
Oceania	13138 (11503-15420)	14.1 (12.3-16.2)	33743 (29408-38878)	13.9 (12.1-15.8)	0.0876 (0.0243-0.1508)
South Asia	419 (311-549)	24.2 (18.0-31.5)	1207 (889-1627)	27.5 (20.7-36.5)	0.5602 (0.4940-0.6265)
Southeast Asia	396269 (344236-449100)	122.4 (105.3-139.3)	1054651 (907435-1219147)	129.5 (112.1-149.5)	0.0254 (-0.0913-0.1423)
Southern Latin America	64130 (55030-74030)	44.3 (37.9-50.6)	135852 (113406-161805)	38.6 (32.4-46.0)	-0.7102 (-0.8217--0.5986)
Southern Sub-Saharan Africa	4621 (4374-4830)	18.3 (17.3-19.1)	7865 (7225-8415)	16.8 (15.5-18.0)	0.2112 (-0.0252-0.4481)
Tropical Latin America	6201 (5133-7363)	36.3 (29.8-43.3)	14606 (12885-16769)	41.0 (36.3-46.8)	0.6459 (0.3588-0.9338)
Western Europe	13796 (13127-14392)	27.1 (25.6-28.4)	36671 (33848-38546)	26.0 (24.0-27.3)	-0.2974 (-0.4418--0.1528)
Western Sub-Saharan Africa	64889 (61781-66697)	22.0 (21.2-22.5)	91803 (82282-97602)	20.8 (19.2-21.8)	-0.0522 (-0.1273-0.0228)
Male					
Global	2021476 (1890182-2164947)	99.18230972 (92.7-106.0)	3866342 (3359573-4249613)	92.1 (80.1-101.1)	-0.3118 (-0.352--0.2716)
High-middle SDI	441463 (422991-462502)	92.4 (88.5-96.6)	607422 (544621-672649)	66.3 (59.5-73.4)	-1.2608 (-1.3401--1.1814)
High SDI	406966 (395136-417536)	84.8 (82.2-87.0)	494715 (473677-515990)	56.9 (54.5-59.3)	-1.2944 (-1.3305--1.2583)
Low-middle SDI	532156 (456088-609659)	148.5 (127.0-170.1)	1226338 (996720-1411527)	158.0 (129.9-181.3)	0.1693 (0.1405-0.1982)
Low SDI	142465 (118094-167504)	106.6 (88.4-125.6)	324414 (258682-387374)	109.8 (88.4-130.7)	-0.0556 (-0.1199-0.0088)
Middle SDI	495927 (454970-541872)	85.0 (78.2-92.7)	1210083 (1034867-1349833)	88.6 (76.0-98.8)	0.1131 (0.0713-0.1548)
Andean Latin America	3200 (2728-3753)	29.1 (24.5-34.3)	6854 (5399-8506)	23.3 (18.4-29.1)	-0.5879 (-0.7400--0.4355)
Australasia	7418 (6700-8149)	68.9 (62.6-75.5)	10416 (9125-11826)	44.9 (39.5-50.8)	-1.3601 (-1.6152--1.1042)
Caribbean	12225 (11173-13598)	95.0 (87.0-105.4)	22243 (18833-26309)	86.6 (73.4-102.2)	-0.0737 (-0.2079-0.0606)
Central Asia	19449 (18098-21006)	87.9 (81.8-95.8)	24511 (21343-28361)	59.6 (52.0-68.8)	-1.2718 (-1.4308--1.1124)
Central Europe	99992 (95232-105268)	147.1 (140.2-154.7)	122849 (112686-133423)	137.9 (126.4-149.9)	-0.3801 (-0.5197--0.2403)
Central Latin America	16922 (16260-17568)	38.9 (37.3-40.4)	37141 (32335-42096)	31.3 (27.2-35.4)	-0.9819 (-1.1005--0.8633)
Central Sub-Saharan Africa	6852 (5015-10184)	56.0 (41.3-81.5)	18340 (13582-24308)	58.6 (44.4-76.1)	0.1199 (-0.0031-0.2430)
East Asia	213931 (176567-255648)	45.2 (37.5-53.7)	537810 (418440-678690)	50.4 (39.6-63.2)	0.6045 (0.4634-0.7458)
Eastern Europe	177426 (169726-190212)	154.9 (148.4-165.8)	198475 (174246-224154)	143.5 (126.1-161.8)	-0.7113 (-0.9161--0.506)
Eastern Sub-Saharan Africa	40054 (33862-46904)	92.8 (78.6-108.0)	90757 (69153-112318)	91.8 (71.2-112.0)	-0.149 (-0.1879--0.1100)
High-income Asia Pacific	35275 (34249-36520)	37.7 (36.5-39.0)	63585 (59033-67230)	35.3 (32.8-37.5)	-0.5945 (-0.949--0.2386)
High-income North America	113973 (111048-116708)	77.7 (75.7-79.5)	132249 (126103-137395)	46.2 (44.2-48.0)	-1.6012 (-1.7812--1.4208)
North Africa and Middle East	22234 (18732-26422)	23.0 (19.3-27.2)	53231 (46117-61103)	20.4 (17.7-23.4)	-0.4565 (-0.4997--0.4133)
Oceania	907 (622-1217)	48.6 (33.7-64.0)	2731 (1905-3605)	56.5 (39.7-74.0)	0.7029 (0.6069-0.7991)
South Asia	773326 (672874-876143)	216.9 (188.3-245.7)	1858748 (1483108-2166194)	229.3 (184.5-266.7)	0.0903 (0.0499-0.1307)
Southeast Asia	110914 (94809-126783)	81.8 (7.0-93.7)	275827 (233449-318325)	82.0 (69.8-94.3)	-0.0379 (-0.0594--0.0163)
Southern Latin America	16787 (14985-18722)	77.6 (69.3-86.4)	17921 (15649-20224)	46.6 (40.8-52.6)	-1.4110 (-1.6583--1.1632)
Southern Sub-Saharan Africa	17725 (13084-21874)	129.8 (94.3-160.6)	34143 (28807-38729)	118.2 (100.8-133.0)	-0.5918 (-0.8527--0.3301)
Tropical Latin America	54472 (51795-57463)	111.9 (105.9-118.0)	117654 (109572-126187)	95.7 (89.2-102.6)	-0.5513 (-0.6904--0.4121)
Western Europe	268219 (256502-278708)	114.2 (109.3-118.8)	214018 (200137-227173)	58.2 (54.6-61.7)	-2.2452 (-2.3112--2.1791)
Western Sub-Saharan Africa	10165 (8026-12399)	19.4 (15.4-23.7)	26828 (20207-34029)	23.1 (17.8-28.9)	0.5209 (0.4409-0.6009)

**Table 6:** Changes in lip and oral cavity cancer YLDs by regions and sexes.

Locations	1990 Number (95% UI, cases)	1990 Age-standardized rate (95% UI, per 100,000)	2021 Number (95% UI, cases)	2021 Age-standardized rate (95% UI, per 100,000)	Estimated annual percentage change (95% CI, 100%)
Both sexes					
Global	64685 (47399-83186)	1.6 (1.1-2.0)	160873 (118043-210399)	1.9 (1.4-2.4)	0.5507 (0.4933-0.6081)
High-middle SDI	13488 (9811-17519)	1.3 (1.0-1.7)	30015 (21934-39119)	1.5 (1.1-2.0)	0.4801 (0.3928-0.5675)
High SDI	25177 (18804-32767)	2.4 (1.8-3.1)	45887 (34195-59876)	2.4 (1.8-3.2)	0.1937 (0.1324-0.2551)
Low-middle SDI	11304 (8374-14840)	1.7 (1.2-2.2)	34038 (24696-45279)	2.2 (1.6-2.9)	0.8412 (0.7418-0.9407)
Low SDI	3008 (2159-3943)	1.2 (0.9-1.6)	8312 (6030-11290)	1.4 (1.0-1.9)	0.4584 (0.3437-0.5732)
Middle SDI	11638 (8433-15167)	1.0 (0.7-1.3)	42487 (30295-56024)	1.5 (1.1-2.0)	1.2793 (1.1687-1.3899)
Andean Latin America	96 (67-125)	0.4 (0.3-0.6)	358 (237-506)	0.6 (0.4-0.8)	1.0618 (0.8929-1.2309)
Australasia	765 (552-1035)	3.3 (2.4-4.5)	1650 (1165-2169)	3.4 (2.4-4.5)	0.0754 (-0.0909-0.2420)
Caribbean	378 (279-493)	1.4 (1.1-1.9)	785 (566-1029)	1.5 (1.1-1.9)	0.3131 (0.2045-0.4218)
Central Asia	444 (321-577)	0.9 (0.6-1.2)	772 (549-1039)	0.9 (0.6-1.2)	0.0051 (-0.2696-0.2805)
Central Europe	2299 (1670-2999)	1.6 (1.1-2.0)	4447 (3262-5844)	2.2 (1.6-2.9)	1.1166 (0.9997-1.2337)
Central Latin America	529 (385-694)	0.6 (0.4-0.9)	1680 (1196-2272)	0.7 (0.5-0.9)	0.1153 (0.0194-0.2112)
Central Sub-Saharan Africa	153 (104-216)	0.6 (0.4-0.9)	451 (303-656)	0.7 (0.5-1.1)	0.4503 (0.2655-0.6355)
East Asia	5695 (3950-7685)	0.6 (0.4-0.8)	25823 (17762-34761)	1.2 (0.8-1.6)	2.4282 (2.2812-2.5755)
Eastern Europe	4275 (3092-5539)	1.5 (1.1-2.0)	7270 (5304-9484)	2.3 (1.6-3.0)	0.9698 (0.7618-1.1783)
Eastern Sub-Saharan Africa	838 (598-1125)	1.0 (0.7-1.4)	2164 (1526-2993)	1.1 (0.8-1.5)	0.1533 (0.0514-0.2553)
High-income Asia Pacific	2275 (1675-2977)	1.1 (0.8-1.5)	6601 (4785-8670)	1.7 (1.2-2.2)	1.3111 (1.008-1.6152)
High-income North America	11067 (8299-14552)	3.3 (2.5-4.7)	17116 (12825-22545)	2.8 (2.1-3.7)	-0.5433 (-0.6226--0.464)
North Africa and Middle East	662 (479-899)	0.4 (0.3-0.5)	2564 (1851-3502)	0.5 (0.4-0.7)	1.2400 (1.1677-1.3124)
Oceania	21 (14-31)	0.6 (0.4-0.9)	66 (44-97)	0.8 (0.5-1.1)	0.7034 (0.6023-0.8045)
South Asia	15812 (11498-20715)	2.4 (1.7-3.1)	51455 (36749-67858)	3.2 (2.3-4.2)	0.8283 (0.6997-0.9570)
Southeast Asia	3244 (2322-4331)	1.2 (0.7-1.6)	10550 (7625-14134)	1.5 (1.1-2.1)	0.7181 (0.656-0.7802)
Southern Latin America	499 (353-663)	1.1 (0.8-1.4)	864 (616-1164)	1.0 (0.7-1.3)	0.1042 (-0.1187-0.3276)
Southern Sub-Saharan Africa	386 (262-533)	1.3 (0.9-1.8)	877 (634-1164)	1.4 (1.0-1.8)	0.0851 (-0.0221-0.1924)
Tropical Latin America	1177 (862-1521)	1.2 (0.9-1.5)	3594 (2608-4715)	1.4 (1.0-1.8)	0.3554 (0.2639-0.4470)
Western Europe	13750 (10161-17969)	2.6 (2.0-3.5)	20804 (15171-27077)	2.6 (1.9-3.4)	0.0347 (-0.0451-0.1145)
Western Sub-Saharan Africa	310 (222-421)	0.3 (0.2-0.4)	971 (679-1363)	0.4 (0.3-0.6)	0.9009 (0.8225-0.9793)
Female					
Global	20918 (15331-27162)	1.0 (0.7-1.2)	57404 (42494-75994)	1.3 (0.9-1.7)	0.8532 (0.7828-0.9236)
High-middle SDI	3451 (2490-4610)	0.6 (0.4-0.8)	9590 (6970-12721)	0.9 (0.8-1.2)	1.3445 (1.2666-1.4225)
High SDI	7790 (5764-10292)	1.3 (0.9-1.7)	15696 (11636-20912)	1.5 (1.1-2.1)	0.6858 (0.6129-0.7588)
Low-middle SDI	4431 (3116-5779)	1.3 (0.9-1.7)	14591 (10387-19621)	1.8 (1.3-2.4)	0.9786 (0.8613-1.0960)
Low SDI	1211 (838-1637)	1.0 (0.7-1.3)	3567 (2562-4870)	1.2 (0.9-1.6)	0.5484 (0.4155-0.6814)
Middle SDI	4014 (2887-5276)	0.7 (0.5-0.9)	13917 (9944-18535)	1.0 (0.7-1.3)	0.8959 (0.7613-1.0307)
Andean Latin America	45 (33-60)	0.4 (0.3-0.5)	192 (130-270)	0.6 (0.4-0.9)	1.3518 (1.1792-1.5247)
Australasia	239 (170-323)	1.9 (1.4-2.6)	556 (393-753)	2.1 (1.5-2.9)	0.4530 (0.2501-0.6563)
Caribbean	117 (86-152)	0.8 (0.6-1.1)	224 (157-307)	0.8 (0.6-1.1)	-0.0349 (-0.1651-0.0956)
Central Asia	147 (104-194)	0.5 (0.4-0.7)	316 (218-439)	0.7 (0.5-0.9)	0.8189 (0.4924-1.1465)
Central Europe	526 (385-699)	0.6 (0.5-0.9)	1250 (928-1652)	1.1 (0.8-1.5)	1.8697 (1.7867-1.9528)
Central Latin America	224 (166-292)	0.5 (0.4-0.6)	769 (549-1048)	0.6 (0.4-0.8)	0.3752 (0.2618-0.4887)
Central Sub-Saharan Africa	64 (43-93)	0.5 (0.3-0.7)	191 (115-307)	0.6 (0.3-0.9)	0.4323 (0.2820-0.5829)
East Asia	2081 (1446-2803)	0.4 (0.3-0.6)	6821 (4507-9544)	0.6 (0.4-0.9)	1.0169 (0.8483-1.1859)
Eastern Europe	1109 (797-1471)	0.7 (0.5-0.9)	2424 (1760-3284)	1.3 (0.9-1.7)	2.1236 (1.8549-2.3930)
Eastern Sub-Saharan Africa	322 (224-437)	0.8 (0.5-1.0)	861 (603-1198)	0.8 (0.6-1.1)	0.1284 (-0.0047-0.2615)
High-income Asia Pacific	852 (618-1126)	0.8 (0.6-1.0)	2583 (1843-3499)	1.2 (0.8-1.6)	1.4833 (1.1693-1.7983)
High-income North America	3888 (2880-5118)	2.0 (1.5-2.7)	5880 (4399-7760)	1.8 (1.3-2.4)	-0.4753 (-0.5674--0.3832)
North Africa and Middle East	267 (189-363)	0.3 (0.2-0.4)	1122 (804-1583)	0.5 (0.3-0.7)	1.6728 (1.5645-1.7812)
Oceania	8 (5-12)	0.5 (0.3-0.7)	24 (16-35)	0.6 (0.4-0.8)	0.5553 (0.4520-0.6587)
South Asia	5770 (4100-7533)	1.8 (1.3-2.4)	20535 (14811-27535)	2.5 (1.8-3.4)	0.9211 (0.7554-1.0871)
Southeast Asia	1439 (1030-1892)	1.0 (0.7-1.3)	4284 (3046-5696)	1.2 (0.9-1.6)	0.3454 (0.2535-0.4375)
Southern Latin America	120 (83-158)	0.5 (0.3-0.6)	296 (213-395)	0.6 (0.5-0.8)	1.3881 (1.1322-1.6447)
Southern Sub-Saharan Africa	115 (81-161)	0.7 (0.5-1.0)	304 (220-403)	0.9 (0.6-1.1)	0.9308 (0.7521-1.1099)
Tropical Latin America	283 (206-373)	0.6 (0.4-0.7)	1035 (747-1361)	0.7 (0.5-1.0)	0.662 (0.5437-0.7804)
Western Europe	3118 (2290-4109)	1.0 (0.8-1.4)	7145 (5273-9554)	1.6 (1.2-2.2)	1.7274 (1.5516-1.9035)
Western Sub-Saharan Africa	174 (119-241)	0.4 (0.3-0.5)	581 (400-833)	0.5 (0.3-0.7)	0.9196 (0.8629-0.9764)
Male					
Global	43767 (32062-55845)	2.2 (1.6-2.8)	103468 (74778-135007)	2.5 (1.8-3.2)	0.3842 (0.3258-0.4427)
High-middle SDI	10036 (7262-12946)	2.2 (1.6-2.8)	20424 (14902-26931)	2.2 (1.6-3.0)	0.0839 (-0.0069-0.1748)
High SDI	17387 (12949-22598)	3.6 (2.7-4.7)	30191 (22495-39272)	3.4 (2.5-4.5)	-0.1142 (-0.1708--0.0576)
Low-middle SDI	6873 (4948-9169)	2.0 (1.4-2.6)	19446 (13485-26219)	2.6 (1.8-3.4)	0.7941 (0.7045-0.8838)
Low SDI	1797 (1266-2471)	1.4 (2.0-1.9)	4744 (3326-6475)	1.7 (1.2-2.3)	0.4251 (0.3211-0.5293)
Middle SDI	7623 (5464-9960)	1.4 (1.0-1.8)	28570 (20188-38092)	2.1 (1.5-2.8)	1.5119 (1.3985-1.6255)
Andean Latin America	50 (34-67)	0.5 (0.3-0.6)	165 (107-234)	0.6 (0.4-0.8)	0.7623 (0.5731-0.9519)
Australasia	526 (377-717)	4.9 (3.5-6.6)	1093 (742-1496)	4.7 (3.2-6.4)	-0.1186 (-0.276-0.0391)
Caribbean	261 (192-346)	2.0 (1.5-2.7)	560 (401-744)	2.2 (1.6-2.9)	0.4831 (0.3448-0.6216)
Central Asia	297 (214-385)	1.4 (1.0-1.8)	455 (322-623)	1.2 (0.8-1.6)	-0.5130 (-0.7779--0.2474)
Central Europe	1773 (1291-2318)	2.6 (1.9-3.4)	3197 (2336-4222)	3.5 (2.6-4.6)	0.8323 (0.6911-0.9738)
Central Latin America	304 (220-401)	0.7 (0.5-1.0)	910 (639-1232)	0.8 (0.5-1.1)	-0.0501 (-0.1610-0.0609)
Central Sub-Saharan Africa	88 (55-137)	0.8 (0.5-1.2)	260 (169-375)	0.9 (0.6-1.3)	0.4817 (0.2837-0.6801)
East Asia	3614 (2504-4932)	0.8 (0.5-1.1)	19002 (12778-26105)	1.8 (1.2-2.4)	3.0525 (2.8699-3.2354)
Eastern Europe	3165 (2305-4104)	2.8 (2.0-3.6)	4846 (3432-6379)	3.5 (2.5-4.6)	0.4724 (0.2872-0.6579)
Eastern Sub-Saharan Africa	516 (366-698)	1.3 (0.9-1.7)	1303 (896-1842)	1.4 (1.0-1.9)	0.2334 (0.1530-0.3139)
High-income Asia Pacific	1422 (1039-1847)	1.5 (1.1-2.0)	4018 (2911-5258)	2.2 (1.6-2.9)	1.1186 (0.8210-1.4171)
High-income North America	7178 (5379-9471)	4.8 (3.6-6.4)	11235 (8360-14729)	3.9 (2.9-5.1)	-0.6398 (-0.7302--0.5492)
North Africa and Middle East	394 (281-534)	0.4 (0.3-0.6)	1441 (1027-1975)	0.6 (0.4-0.8)	0.9217 (0.8663-0.9772)
Oceania	13 (8-19)	0.7 (0.5-1.1)	41 (27-62)	0.9 (0.6-1.4)	0.7902 (0.6888-0.8917)
South Asia	10042 (7133-13333)	2.9 (2.1-3.9)	30919 (20956-41641)	3.9 (2.7-5.2)	0.8407 (0.7358-0.9457)
Southeast Asia	1805 (1266-2414)	1.4 (1.0-1.8)	6265 (4483-8462)	1.9 (1.4-2.5)	0.9882 (0.9359-1.0404)
Southern Latin America	379 (264-506)	1.8 (1.2-2.4)	567 (396-768)	1.5 (1.0-2.0)	-0.4149 (-0.6425--0.1869)
Southern Sub-Saharan Africa	270 (174-385)	2.0 (1.3-2.9)	573 (407-775)	2.0 (1.5-2.7)	-0.2540 (-0.3898--0.1179)
Tropical Latin America	894 (655-1149)	1.9 (1.4-2.4)	2558 (1866-3344)	2.1 (1.5-2.7)	0.2982 (0.1985-0.3981)
Western Europe	10632 (7818-13717)	4.5 (3.3-5.8)	13659 (9911-17862)	3.7 (2.7-4.8)	-0.6584 (-0.7233--0.5934)
Western Sub-Saharan Africa	135 (95-187)	0.3 (0.2-0.4)	389 (268-556)	0.4 (0.2-0.5)	0.7845 (0.6760-0.8932)

**Figure 1:**
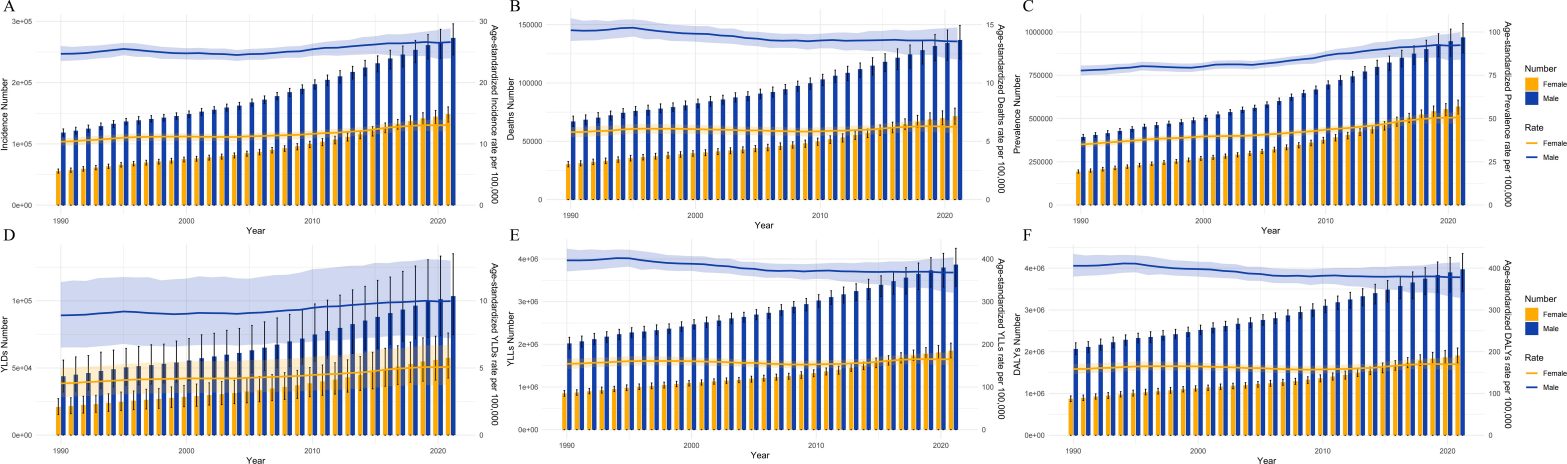
Trends in the disease burden of lip and oral cavity cancer (LOCC) by gender from 1990 to 2021. **(A–F)** represent the incidence, death, prevalence, years lived with disability (YLDs), years of life lost (YLLs) and disability-adjusted life years (DALYs), respectively.

Regarding the trends in changes, from 1990 to 2021, ASIR, ASPR, and ASR of YLDs for LOCC exhibited an estimate annual increasing trend (EAPC, 0.3981 [95% CI, 0.3332 to 0.4631]; 0.8167 [95% CI, 0.7644 to 0.8690]; 0.5507 [95% CI, 0.4933 to 0.6081]) globally (**Figures 1A, C, F**). Among the 21 GBD regions, East Asia had the highest estimate annual increase in ASIR, ASPR, and ASR of YLDs (EAPC, 1.8681 [95% CI, 1.7032 to 2.0333]; 3.3177 [95% CI, 3.1823 to 3.4533]; 2.4282 [95% CI, 2.2812 to 2.5755]) (**Table 1**–**6**). In contrast to the aforementioned trends, the ASDR, ASR of DALYs, and YLLs caused by LOCC all exhibited an estimate annual decreasing trend (EAPC, -0.1142 [95% CI, -0.1675 to -0.0608]; -0.1650 [95% CI, -0.2178 to -0.1121]; -0.1832 [95% CI, -0.2361 to -0.1302]) (**Figures 1B, D, E**). Ultimately, Western Europe exhibited the most substantial estimate annual decline in ASDR, ASR of DALYs, and YLLs (EAPC, -1.2628 [95% CI, -1.3406 to -1.1850]; -1.6138 [95% CI, -1.6700 to -1.5576]; -1.7016 [95% CI, -1.7595 to -1.6437]). Notably, the region where all LOCC burden indicators showed a marked increase was Oceania.

In terms of sex differences, a notable observation is that among males in East Asia, the highest annual percentage increases were seen in ASIR, ASPR, and the ASR of YLDs (EAPC, 2.4966 [95% CI, 2.3051 to 2.6885]; 4.0502 [95% CI, 3.8578 to 4.2428]; 3.0525 [95% CI, 2.8699 to 3.2354]). Conversely, among females in East Asia, ASPR, ASDR, the ASR of DALYs, and YLLs all exhibited a marked annul decreasing trend (EAPC, -1.9484 [95% CI, -2.1384 to -1.7581]; -1.7080 [95% CI, -1.8764 to -1.5394]; -1.8521 [95% CI, -2.0437 to -1.6601]; -1.9484 [95% CI, -2.1384 to -1.7581]) (**Table 1**-**6**).

### Projections of the burden of LOCC from 2022 to 2030

**Figure 2:**
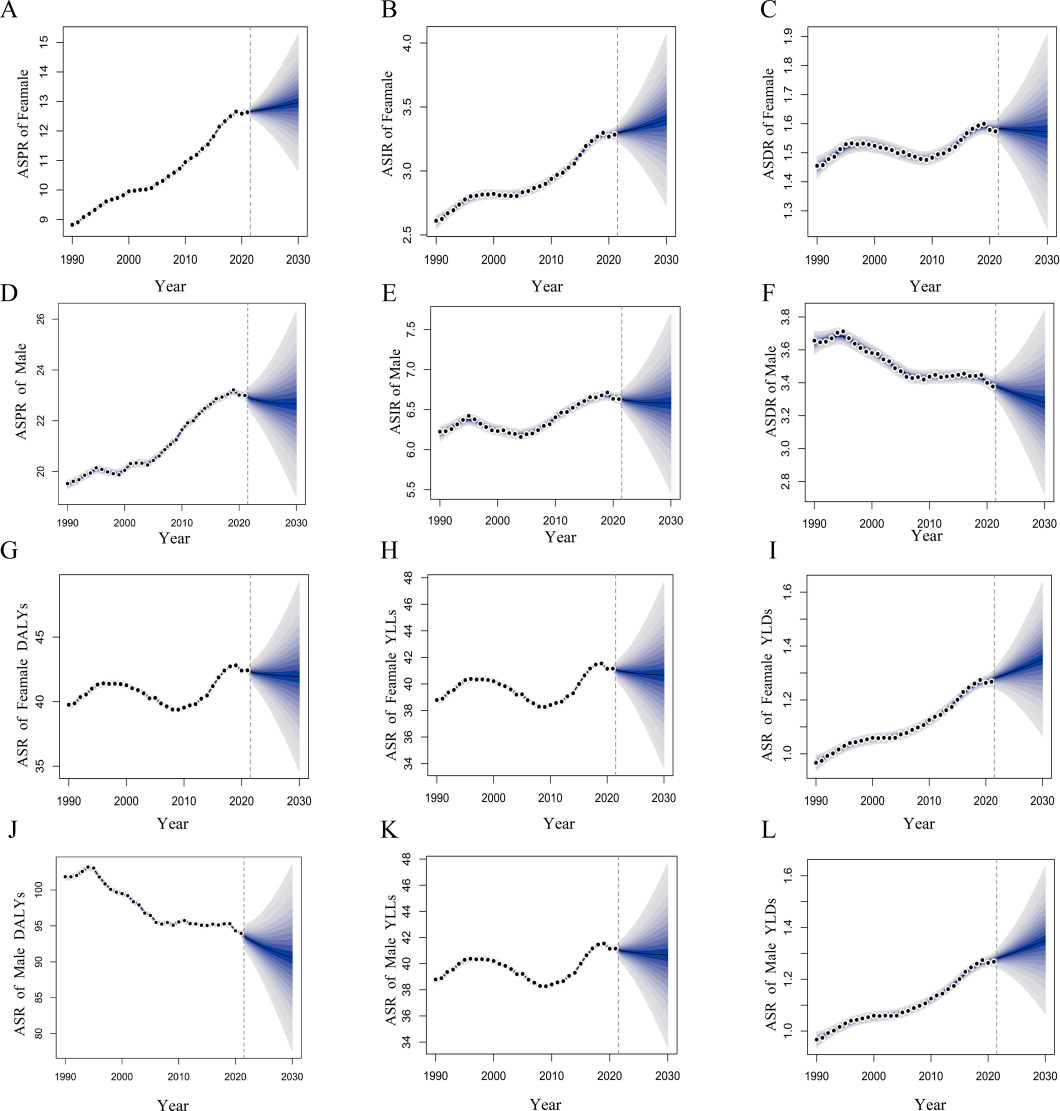
Projections of the burden of lip and oral cavity cancer (LOCC) by gender from 2022 to 2030 based on the Bayesian Age-Period-Cohort (BAPC) model. **(A–C)** depict the forecasted age-standardized prevalence rate (ASPR), age-standardized incidence rate (ASIR), and age-standardized death rate (ASDR) for females, respectively. **(D–F)** present the corresponding projections for males. **(G-I)** illustrate the predicted age-standardized rates of disability-adjusted life years (DALYs), years of life lost (YLLs), and years lived with disability (YLDs) for females, while Panels **(J-L)** show these predictions for males.

Based on the BAPC predictive model, this study found that the global burden of LOCC will exhibit significant sex differences from 2022 to 2030. Specifically, the ASIR and ASPR among males are projected to decline, whereas the opposite trend is anticipated for females. Moreover, by 2030, both sexes are expected to experience a decrease in the ASDR, ASR of DALYs, and YLLs. However, the ASR of YLDs is projected to increase for both sexes (**Figure 2**).

Specifically, compared to the Incidence of LOCC in males in 2021, the number of cases or years is projected to increase by 17.3% by 2030, the number of Death by 12.1%, the Prevalence by 17.4%, the number of DALYs by 14.7%, the number of YLLs by 14.6%, and the number of YLDs by 9.1%. Except for the ASR of YLDs in males, all other indicators were expected to show a downward trend. Specifically, the ASIR was projected to decrease from 6.65 to 6.58, the ASDR from 3.39 to 3.28, the ASPR from 23.02 to 22.66, the ASR of DALYs from 93.98 to 90.58, and the ASR of YLLs from 91.50 to 88.16, while the ASR of YLDs is expected to increase from 2.49 to 2.51, with all rates expressed per 100,000 population. Additionally, by 2030, the Incidence of LOCC in females was projected to increase by 18.3%, Death by 7.9%, Prevalence by 23.2%, DALYs by 18.6%, YLLs by 18.2%, and YLDs by 5.7%. The ASIR in females was expected to rise from 3.29 to 3.39, the ASPR from 12.65 to 12.96, and the ASR of YLDs from 1.28 to 1.35. Conversely, the ASDR is projected to decrease from 1.58 to 1.57, the ASR of DALYs from 42.43 to 41.93, and the ASR of YLLs from 41.16 to 40.65, with all rates expressed per 100,000 population. These projections suggest that, compared to 2021, the life milestones of LOCC patients of both sexes in 2030 will likely see a delay in the time of death and an increase in survival duration.

**Figure 3:**
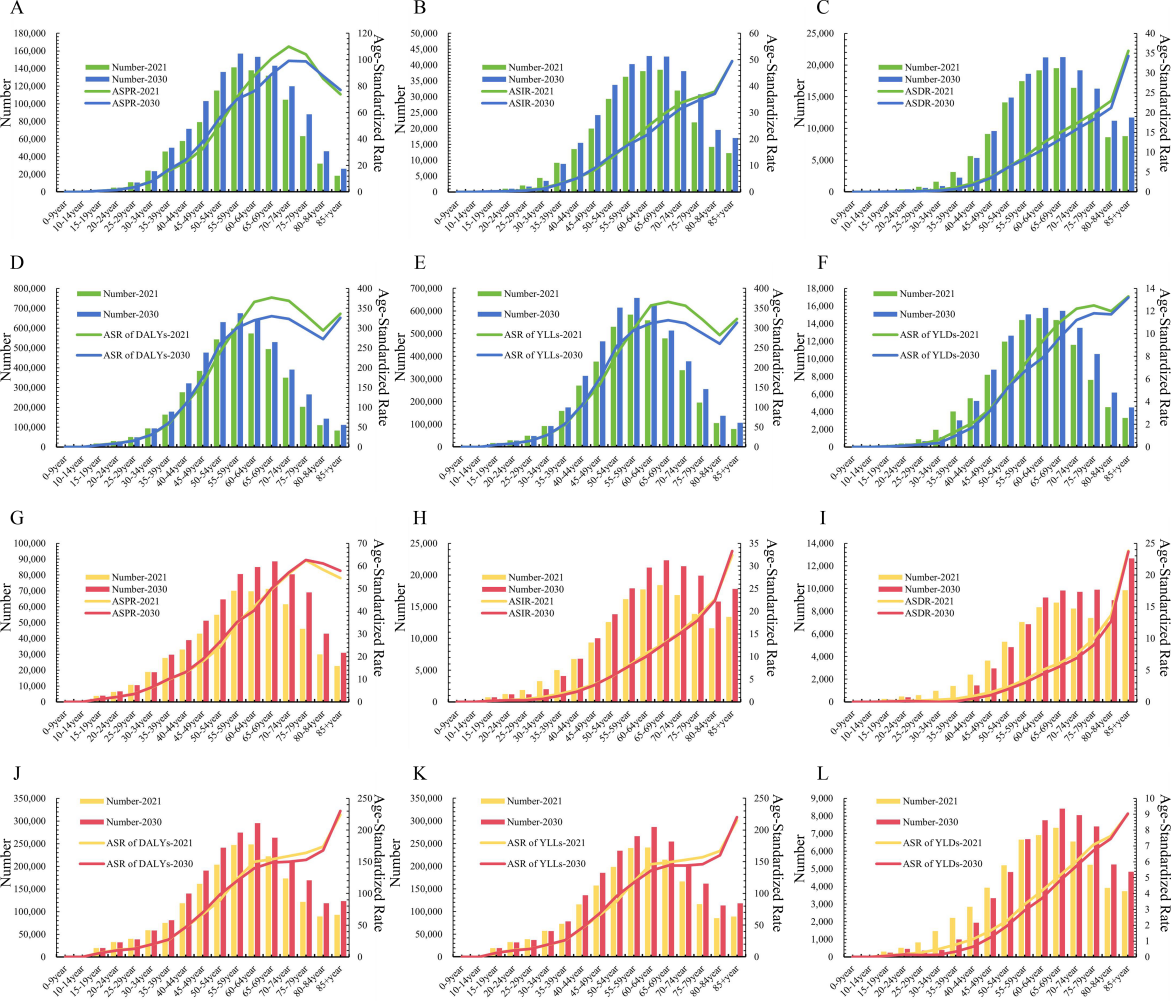
Comparison of projected burden of lip and oral cavity cancer (LOCC) across different age groups for the years 2022 and 2030 based on the Bayesian Age-Period-Cohort (BAPC) model. **(A–F)** represent the prevalence, incidence, deaths, disability-adjusted life years (DALYs), years of life lost (YLLs), and years lived with disability (YLDs) for males, respectively. Panels **(G-L)** depict the corresponding metrics for females.

Moreover, this study also compared the LOCC disease burden among different age groups of males and females in 2021 and 2030. The findings revealed that, for both sexes, the ASR and the number of cases increase with age, particularly in middle-aged and elderly populations, where this trend is more pronounced. Specifically, patients aged over 50 years are projected to constitute the majority of all LOCC patients (**Figure 3**). This indicates that middle-aged and elderly LOCC patients will continue to pose a significant challenge in the efforts to reduce the LOCC disease burden in the future.

## Discussion

Drawing on the GBD 2021 database, this study conducted a analysis of the global burden of LOCC from 1990 to 2021 and employed the BAPC model to forecast the trends of LOCC from 2022 to 2030. Globally, South Asia emerged as the GBD region with the most severe LOCC burden in 2021. Between 1990 and 2021, the global ASIR, ASPR, and ASR of YLDs for LOCC exhibited a marked upward trend, whereas the ASDR, ASR of DALYs, and ASR of YLLs showed a downward trajectory. Notably, East Asia and Western Europe experienced more pronounced annual changes in LOCC burden. By 2030, the DALYs and YLLs are projected to decline, likely due to a reduction in death and an extension in survival duration among LOCC patients, while the ASR of YLDs is expected to rise. Overall, the burden of LOCC remains a significant global concern.

Numerous factors contribute to the severe burden of LOCC. Among these, lifestyle habits exert a particularly significant influence on LOCC (15). Specifically, the practice of chewing betel quid can directly damage the oral mucosa and induce genetic mutations, thereby triggering oral cavity cancer (15, 16). China, Myanmar, India, and Bangladesh, as major global producers of betel nut, also exhibit extremely high levels of betel nut consumption (17, 18). Moreover, approximately half of the oral cavity cancer cases reported in East Asian countries are attributed to betel nut chewing (19). This is likely one of the reasons for the prominent EAPC of LOCC in East Asia and the ASDR in South Asia. Furthermore, ultraviolet (UV) radiation serves as a critical risk factor for the development of lip cancer (20). UV rays have the capacity to disrupt DNA within skin and mucosal cells, thereby inducing genetic mutations and elevating the likelihood of lip cancer (21, 22). Our research has identified a marked escalation in the burden of LOCC within Oceania, a trend potentially linked to the region’s geographical context. The disparity in solar radiation exposure across regions, attributable to their latitudinal positions and variations in the ozone layer, plays a significant role (23). Regions including South Asia, Oceania, and Australasia et.al, are commonly subjected to higher intensities of UV radiation, which is likely a principal factor underlying the substantial burden of lip cancer in these locales (24, 25). Consequently, the adoption of prudent sun protection strategies is instrumental in mitigating the risk of lip cancer. It is essential to initiate preventive initiatives aimed at enhancing the awareness of utilizing sunscreen and hats, especially within countries with strong UV.

The global ASDR of LOCC has shown a gradual decline (with a negative EAPC), and projections for 2022–2030 indicate a downward trend in the ASDR, ASR of DALYs, and YLLs due to LOCC worldwide. These positive shifts are likely due to significant advancements in the global LOCC healthcare sector. In terms of early diagnostic techniques, oral exfoliative cytology enables the detection of potential cancer cells in the oral cavity by analyzing cells collected from the oral mucosa, particularly in patients with submucosal fibrosis (10). Innovations in surgical techniques have also led to improved treatment outcomes for LOCC patients (26, 27). Targeted radiotherapy technologies can precisely focus high doses of radiation on tumor sites, enhancing local tumor control rates and improving the survival duration of LOCC patients (28–31). However, there were notable differences in the changes of ASDR across different regions. Western Europe has experienced the most significant average annual decline in ASDR, with a marked decrease observed in males, which may be associated with its comprehensive and advanced healthcare system. In Western Europe, efficient cancer screening networks have been established, facilitating more accurate early detection of cancer (3, 32). Meanwhile, the decline in the burden of LOCC in Australasia and High-income North America, including Australia and the United States, may be related to a decrease in the number of people working outdoors and an increase in primary prevention of solar radiation by outdoor workers using sunscreen and protective clothing and headgear (4). Therefore, we advocate that early screening for LOCC should draw inspiration from Western Europe, aiming to refine and establish an effective early detection system for cancers.

This study projects that between 2022 and 2030, the ASIR and ASPR of LOCC will exhibit a declining trend in males globally, whereas an upward trend is anticipated in females. Moreover, the burden of LOCC is particularly severe among middle-aged and elderly populations, with older females being disproportionately affected. This phenomenon may be closely linked to a lack of awareness regarding oral health among the elderly (33). Many seniors fail to implement timely and effective personal oral care in their daily routines, leading to a gradual accumulation of oral health issues (34, 35). the phenomenon of delayed medical treatment within the elderly population also contributes to the failure to effectively control oral diseases at early stages, thereby exacerbating the overall LOCC burden (34). As the population continues to age, the LOCC burden among middle-aged and elderly individuals will face even more formidable challenges in the future. Furthermore, the ASIR and ASPR of female LOCC were higher than those of males, which may be attributed to changes in consumption patterns of cigarette smoking (4). Scholars have pointed out that the impact of smoking on the historical trends of oral cavity cancer is reflected in its similarity to the trends of lung cancer two decades later; in several European countries, the incidence of lung cancer among male continues to decline, while it rises among female, which coincides with the lag time of cigarette smoking consumption (4). Consequently, there is an urgent need to develop more targeted diagnostic and treatment measures, enhance educational efforts on lip and oral cavity health for the elderly, elevate their awareness of lip and oral cavity health, and optimize the allocation of medical service resources to address the growing burden of LOCC. At the same time, it is also necessary to strengthen publicity about the hazards of smoking consumption to LOCC and raise public awareness regarding this risk factor.

From a disease control and prevention strategy perspective, the findings of this study provide direction for global LOCC management. For regions experiencing rapid increases in Incidence rates, such as East Asia, it is essential to widely disseminate health knowledge related to LOCC and enhance the public’s awareness of the disease. Concurrently, establishing a robust screening system, increasing investment in oral healthcare facilities, training specialized oral screening personnel, and promoting regular oral examinations are crucial, especially for high-risk groups such as long-term betel nut chewers, who should have shorter intervals between check-ups to facilitate early detection and treatment. For regions where the ASDR was not significantly declining or still rising, such as Oceania and some parts of Sub-Saharan Africa, in addition to strengthening health education and screening efforts, we advocate for increased investment in medical resources, improving primary healthcare facilities, and introducing advanced oral examination equipment and LOCC treatment technologies to areas with a high burden of LOCC.

Lastly, this study also has certain limitations. Issues such as non-standardized data collection methods and insufficient sample representativeness may lead to some variation in data quality, potentially interfering with the accuracy of the analytical results to a certain extent. For instance, some developing countries may experience underreporting or misreporting due to a lack of comprehensive disease registration systems and professional data collectors, affecting an accurate assessment of the local LOCC burden. Thus, we suggest integrating and strengthen cancer registry data to mitigate this limitation in future studies. Furthermore, as this study was based on existing data and models for analysis and forecasting, there may be unforeseen factors impacting the burden of LOCC.

## Conclusion

The LOCC burden remains severe at present, but it is anticipated to exhibit a downward trend by 2030. Consequently, it is imperative to intensify efforts in the prevention and control of LOCC to meet these expectations. This study offers theoretical guidance for mitigating the burden of LOCC. In light of the findings, we should enhance control and preventive measures against LOCC, focusing on aspects such as regulating betel nut consumption, promoting lip and oral cavity sun protection, and ensuring timely access to medical services. These targeted interventions are crucial for reducing the incidence and improving the prognosis of LOCC, thereby contributing to a decrease in the overall disease burden. By prioritizing these areas, we can work towards a future where the impact of LOCC on global health is significantly diminished.

## Data Availability

Publicly available datasets were analyzed in this study. This data can be found here: https://vizhub.healthdata.org/gbd-results/.
